# Reconstructive Surgery for Head and Neck Cancer Patients

**DOI:** 10.1155/2014/795483

**Published:** 2014-11-09

**Authors:** Matthew M. Hanasono

**Affiliations:** The University of Texas M.D. Anderson Cancer Center, 1515 Holcombe Boulevard, Unit 443, Houston, TX 77030, USA

## Abstract

The field of head and neck surgery has gone through numerous changes in the past two decades. Microvascular free flap reconstructions largely replaced other techniques. More importantly, there has been a paradigm shift toward seeking not only to achieve reliable wound closure to protect vital structures, but also to reestablish normal function and appearance. The present paper will present an algorithmic approach to head and neck reconstruction of various subsites, using an evidence-based approach wherever possible.

## 1. Introduction

The field of head and neck reconstructive surgery is a dynamic one. Advances made in the last decade are mostly secondary to expanded use of microvascular free flaps [1]. Several flaps, including the anterolateral thigh, fibula osteocutaneous, and suprafascial radial forearm fasciocutaneous free flaps, have emerged as workhorse flaps for reconstructing a wide variety of defects. As the anatomy of these flaps has become more familiar, their reliability and versatility have increased. Reliable wound closure without exposure of vital structures is no longer the only priority. Preserving function, including speech and swallowing, and restoring appearance are the goals in every reconstruction. Free flap success rates now routinely exceed 95 percent or better at most centers [1–3]. On top of this, minimizing flap donor site morbidity is an important consideration. Because of the high rate of recurrence as well as long-term complications following major head and neck resections and reconstructions, preservation of recipient vessel options and flap donor sites should also be a consideration. In the following paper, an algorithmic approach to mid-facial, mandibular, oral cavity, and pharyngoesophageal reconstruction will be reviewed and expected outcomes discussed.

## 2. Mid-Facial Reconstruction

Management of mid-facial defects is among the most complicated and controversial areas of head and neck oncologic reconstruction. Options include use of prosthetic obturators, pedicled flaps, and free flaps, sometimes combined with grafts or alloplasts [4]. The popularity of pedicled flaps has declined in recent years due to limited reach and volume. Prosthetic obturators remain a good solution for some patients with limited defects. For extensive defects, obturators may be difficult or impossible to retain, particularly in edentulous patients [5]. Furthermore, obturators are inappropriate for defects that involve resection of the skull base, orbital contents, orbital floor, or soft tissues of the face. Finally, some patients may not like the inconvenience of an obturator, which must be removed and cleaned regularly and periodically adjusted or replaced for fit and/or hygiene.

Mid-facial reconstructions with various bony and soft tissue free flaps have been described, and the best technique remains a subject of debate [6–11]. One of the fundamental problems with reconstructing the mid-face is that the defects created by oncologic resection are highly variable. Such defects usually not only involve the maxillary bones, but also may include a number of adjacent facial and cranial bones, as well as soft tissues of the face, palate, and orbit. Successful outcomes in mid-facial reconstruction involve not only a mastery of a broad range of reconstructive flaps and craniofacial plating techniques, but also an understanding of the requirements for prosthetic rehabilitation, which is used not only in place of reconstruction in some cases, but also often in concert with local and distant tissue transfer procedures.

### 2.1. Regional Anatomy and Nomenclature

The central structures of the mid-face are the paired maxillary bones, which are fused in the midline to form the upper jaw. The maxillary bones contribute to the roof of the mouth, the floor and lateral wall of the nasal cavity, and the floor and medial walls of the orbit. Each maxilla attaches laterally to the zygomatic bones, which comprise part of the orbital floor and the lateral orbital wall and provide shape to the cheek. In addition to the zygomatic bones, the maxillae also articulate with the frontal and ethmoid bones of the cranium and the nasal, lacrimal, inferior nasal conchal, palatine, and vomer bones of the face.

Many maxillary tumors extend into or arise from the orbit, which is a conical structure that contains the eye, extraocular muscles, and extraocular fat, as well as blood vessels and cranial nerves II, III, IV, V, and VI. The superior margin of the orbit is the frontal bone, the inferior margin is the maxilla, palatine, and zygomatic bones, the medial margin is the frontal, lacrimal, and ethmoid bones, and the lateral margin is the zygomatic and sphenoid bones. The orbit lies below the anterior cranial fossa, above the maxillary sinus, lateral to the nasal cavity, and anterior to the middle cranial fossa (medially) and the temporal fossa (laterally). From the orbital rim, the orbit tapers posteriorly to an apex, the entrance of the optic canal. Two large discontinuities, the superior and inferior orbital fissures, converge upon one another in the back of the orbit just lateral to the apex.

Oncologic resections of maxillary tumors can be quite variable [12]. There is no consensus in the literature on the nomenclature of types of maxillectomy. In many publications the term “partial” and the term “subtotal” have been used interchangeably. Spiro et al. [12] divided maxillectomies into limited, subtotal, and total depending on whether the resection involved predominantly one wall, at least two walls including the palate, or the entire maxilla. Others subclassify partial maxillectomy into infrastructure (where only the upper alveolus and hard palate below the level of nasal floor are removed), medial (where medial wall of maxilla often along with the medial 1/3rd of inferior orbital wall and the medial orbital wall is removed), suprastructure (where all the walls of maxilla, except for hard palate and upper alveolus, are removed), and subtotal (where all the walls of maxilla, except for the orbital floor and the zygomatic buttress, are removed) [13].

Orbital exenteration involves removal of all the orbital contents, in contrast to enucleation, which involves removal of only the globe. This technique is used for many adnexal cancers involving the eyelid with orbital extension. When the eyelid skin and orbicularis muscles are not involved in the cancerous process, such as in some palpebral conjunctival and orbital cancers, the anterior lamella of the eyelid (skin or musculocutaneous layer) can be spared and used for coverage of exenterated orbital defect. As a matter of aesthetic preference, the eyelids are still removed by some surgeons and replaced by skin from the reconstructive flap. In extended orbital exenteration, cancers of the paranasal sinuses, nasal cavity, and periorbital and facial soft tissues extending to the orbit require more radical surgical ablation including one or more orbital bony walls, as well as other structures such as the sinuses and facial skin. Both total maxillectomy and suprastructure maxillectomy may be combined with orbital exenteration, technically making it an extended orbital exenteration but more often referred to as an orbitomaxillectomy by most surgeons.

### 2.2. Reconstructive Approach

Medial maxillectomy involves resection of the medial wall of the maxilla and inferior turbinate. This surgery is usually indicated for benign or low-grade tumors arising from the lateral nasal wall, formerly performed through a lateral rhinotomy incision, and it is now frequently performed endoscopically. If no other structures are removed, reconstruction is unnecessary. For the remainder of maxillary and orbital resections, reconstruction or rehabilitation must be addressed with flaps, grafts, and/or prosthetics.

In terms of reconstruction, there are several key considerations. The status of the palate is the main determinator of which flap type, if any, is best suited for reconstructing the defect [5]. The amount of hard and soft palate resected as well as the location of the resection and the plans for dental restoration will dictate whether a prosthetic obturator is indicated or a bony or soft tissue flap should be performed. The status of the orbital floor is important if the orbital contents are to be preserved. Accurate reconstruction here, with grafts, implants, or bony flaps, is mandatory for useful eye function. If an extended orbital exenteration or orbitomaxillectomy is performed, a pedicled or free flap may be indicated to separate the orbit from the nasal cavity and sinuses or occasionally the intracranial cavity. A pedicled or free flap may also be needed to serve as lining of the remaining orbit for an orbital prosthetic when one is desired by the patient. A final consideration is whether facial skin and soft tissues, such as the lips, eyelids, or nose, will be included in the resection. Facial skin may be reconstructed with local tissues (e.g., cervicofacial rotation flap) or a pedicled or free flap, while other facial structures are usually addressed separately, most commonly with local tissue techniques (e.g., paramedian forehead flap for nasal reconstruction).

#### 2.2.1. Suprastructure Maxillectomy

Suprastructure maxillectomies result in defects that do not involve the palate. Suprastructure defects that do not violate the bony orbit do not necessarily need reconstruction. An exception is when facial soft tissues are included in the resection and soft tissue cheek reconstruction is needed. Another exception may be when intracranial contents at the skull base have been exposed. In the latter case, a bulky soft tissue free flap that obliterates the maxillary sinus is recommended to isolate the intracranial cavity from the nasal cavity by creating a watertight seal against the dura or brain, thereby preventing cerebrospinal fluid leaks and meningitis, although small defects can sometimes be sealed with local or pedicled flaps, such as the temporoparietal fascia flap.

#### 2.2.2. Unilateral Posterior Palatomaxillectomy

While any number of palatoalveolar defects is possible, Okay et al. [5] have recommended distinguishing defects based on whether function can be satisfactorily restored with an obturator or if a free flap is required. Palatoalveolar defects that spare both canine teeth can often be successfully treated with an obturator. In these cases, cantilever forces resulting in unstable prosthetic retention are minimized because of the favorable root morphology of the canine adjacent to the obturator and the substantial arch length provided by the remaining alveolus. Thus, defects including unilateral posterior palatomaxillectomy defects or anterior defects limited to the premaxilla, which bears the 4 incisor teeth, can be obturated and should be considered separately from those that cannot, including those that involve half the palate and those that involve the entire anterior arch or whole palate.

Based on this information, unilateral palatomaxillectomy defects posterior to the canine tooth can usually be treated with an obturator. However, some patients may still prefer to undergo autologous reconstruction due to inconvenience, hygiene issues, residual instability, mainly in edentulous patients, and long-term costs associated with periodic adjustment and replacement of the prosthesis [13–15]. Additionally, exposure of the intracranial contents, loss of the orbital floor or orbital contents, and resection of the facial soft tissues are indications for free flap reconstruction. Alternately, a temporalis muscle flap can be tunneled into the defect and placed against the skull base, if exposed, and, if large enough, close the palatal defect [16].

Soft tissue free flaps are our first choice for closure posterior palatomaxillary defects [16–18]. The aesthetic challenge is usually to provide adequate volume to the cheek to support the facial soft tissues and avoid a hollow appearance. An analogous situation is present in the mandible, where posterior mandibular reconstruction with soft tissue flaps can often achieve good results with regard to both function and appearance, provided the flap has adequate bulk. Restoration of posterior maxillary dentition, which is not easily visible even when smiling, is not a priority to many patients.

**Figure 1:**
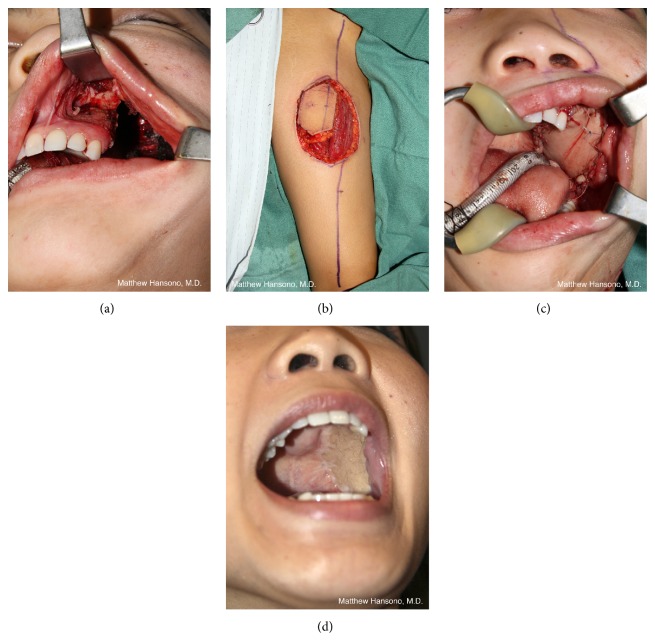
Posterior palatomaxillary defect following tumor removal (a). An anterolateral thigh free flap is harvested, measuring approximately 5 × 5 cm (b). Flap inset (c). Postoperative appearance (d).

The anterolateral thigh (ALT) or rectus abdominis myocutaneous (RAM) free flaps are usually well suited to provide the appropriate amount of tissue for posterior palatomaxillary reconstruction (Figure 1). These flaps tend to be thicker in Western patients and will partially obliterate the maxillary sinus. Both flaps can be dissected such that their muscular components can be minimized and the flaps can be safely defatted in patients with more subcutaneous adipose tissue than desired. By utilizing distal perforators, the pedicle length is usually satisfactory in both flaps to reach the neck blood vessels without need for interposition vein grafting. Suturing to the palatal mucosa should take place over the bony palatal remnant to avoid an oronasal fistula, or holes can be drilled in the bony palate and a deep layer of sutures can be placed through them for an extra degree of wound closure stability.

For all free flap reconstructions in the mid-face, the facial artery and vein or the superficial temporal artery and vein are the preferred recipient vessels when available. When the facial artery and vein are used as recipient blood vessels, a subcutaneous tunnel is created within the cheek to the neck. Care is taken not to injure the parotid duct during tunnel creation by dissecting anterior to the Stensen's duct orifice. Facial nerve injury is avoided by staying within the subcutaneous plane, as in a facelift.

#### 2.2.3. Unilateral Hemipalatomaxillectomy

Unlike unilateral posterior palatomaxillectomy defects, those where the resection of the palate and alveolus extends anterior to the canine tooth defects are difficult to obturate because of the greater cantilever forces acting on the prosthesis, which must also rely on less dentition for retention. Free flap selection for these defects is somewhat controversial. Soft tissue free flaps are usually more straightforward surgically [16–18]. However, they do not provide a rigid skeletal framework, which can result in a loss of anterior maxillary projection on the side of the defect and cannot accept osseointegrated implants for dental restoration. To accommodate a conventional dental prosthesis, the soft tissue flap must not protrude excessively into the oral cavity. However, achieving a concave palatal reconstruction with soft tissue flaps can be technically challenging, especially if the lateral portion of the defect includes some or all of the buccal mucosa.

The author favors the use of osteocutaneous free flaps for hemipalatomaxillectomy defects in highly functional patients with a reasonable oncologic prognosis (Figure 3). Besides providing better anterior projection, osteocutaneous free flaps offer the possibility of osseointegrated implants for dental restoration. A caveat is that postoperative radiation therapy may render placement of osseointegrated implants risky, thus defeating one of the main purposes of bony reconstruction. In such cases, options include initial placement of an obturator, if possible, followed by delayed osteocutaneous free flap reconstruction following the conclusion of radiation or proceeding with immediate bony reconstruction and simultaneous osseointegrated implant placement. Some centers do perform delayed osseointegrated implant placement even into irradiated bony free flaps after treatment with hyperbaric oxygen therapy, although the efficacy of this strategy still needs to be established.

In terms of bony free flap selection, many donor sites have been suggested, including the fibula, scapula, radius, rib, and iliac crest [6–11]. The author favors the fibula because of its high quality bone stock that easily accommodates osseointegrated implants and tolerates the multiple osteotomies necessary to shape the bone so that it resembles the mid-facial form [19]. Regardless of which flap is used, osteotomies should be made to simulate the complex shape of the native maxilla as closely as possible. While it is tempting to simply place vascularized bone in a nonanatomic position and shape, our experience is that the soft tissues of the cheek will eventually contract and reveal the shape of the underlying bone, especially when postoperative radiation is administered.

#### 2.2.4. Bilateral Palatomaxillectomy

Bilateral palatomaxillectomy defects that involve loss of the anterior maxillary alveolar arch, including the canine teeth, need bony reconstruction to maintain mid-facial height, width, and projection. They also require bony reconstruction for dental restoration with osseointegrated implants, which are usually necessary to retain a prosthesis. Bilateral palatomaxillectomy defects can rarely, if ever, be stably obturated [19–21]. Although other osteocutaneous free flaps have been advocated, the fibula free flap is our preferred flap for bilateral palatomaxillectomy reconstruction for the same reasons noted above. In addition, for sizable defects involving both maxillary bones, the fibula offers the longest length of bone of the various flap options.

In our experience, 14 to 16 cm of bone length is typically needed to reconstruct a bilateral maxillectomy defect [19]. The lateral surface of the fibula bone is used to restore the vertical maxillary height, measured from the orbital rim to the occlusal plane of the hard palate, by orienting it such that it faces anteriorly on the face. The peroneal vessels, therefore, assume a posterior position facing the maxillary sinus. Some flexor hallucis longus muscle is usually included with the flap in order to obliterate the maxillary sinus cavity and provide adequate soft tissue around the vascular pedicle to prevent its desiccation. The skin paddle of the flap is used to reconstruct the palatal defect and the nasal surface of the flap is left to mucosalize spontaneously in most cases.

When the bony defect extends more laterally or posteriorly on one side than another, that side is usually preferred for the microvascular anastomosis due to closer proximity to the recipient blood vessels. Alternately, the side with better recipient blood vessels, if any, is selected. The leg that is ipsilateral to the side of the planned microvascular anastomosis is chosen as the fibula osteocutaneous free flap donor side so that the skin paddle can be used to restore the palate with tension on the cutaneous perforators. Vein grafts are used when pedicle length is inadequate to reach the recipient vessels. The facial blood vessels are usually preferred as recipients when available, due to their proximity to the defect and their good size match to the peroneal blood vessels.

**Figure 2:**
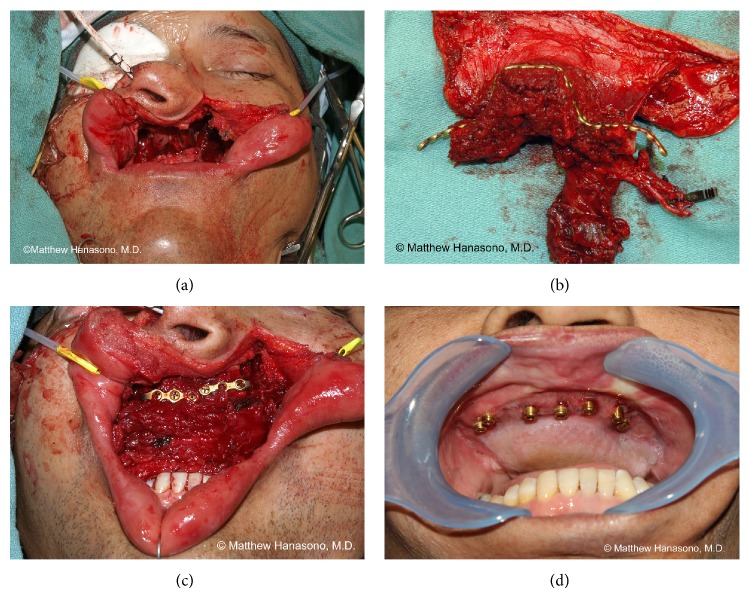
Anterior bilateral palatomaxillary defect following tumor removal (a). A fibula osteocutaneous free flap is osteotomized then rigidly fixated to resemble the Greek letter “omega” in the transverse plane (b). Flap inset (c). Postoperative appearance (d). The skin paddle is used to close the palatal defect, while the bone restores mid-facial height, width, and projection. Osseointegrated implants have been placed.

After the resection is complete, a titanium reconstruction plate is fashioned based on the defect in the approximate shape of the Greek letter “omega” in the transverse plane (Figure 2). The configuration of the reconstruction plate is such that it simulates the width and projection of the native maxilla. The lateral portions of the reconstruction plate must be long enough to allow two or three screw fixations to the remaining zygomatic bones laterally.

Closing wedge osteotomies are performed on the fibular bone with a reciprocating saw, taking care not to injure the vascular pedicle. When reconstructing bilateral maxillectomy defects, the lateral portions of the “omega” recreate the malar regions. The central portion of the fibula free flap restores the maxillary alveolus. For unilateral (see* unilateral hemipalatomaxillectomy*, above) or less than complete bilateral defects, a shorter segment of bone is used and one or more osteotomies can be omitted (i.e., the fibular bone resembles a half or incomplete “omega”).

The portion of the fibula free flap that replaces the anterior maxillae is inset at the vertical level of the resected alveolus, rather than at the level of the dentition, to provide room for an implant-retained dental prosthesis. A slight downward angulation (about 20–25 degrees) of the portion of the fibula used to recreate the anterior maxillae is usually desirable to fully restore vertical facial height [22, 23].

Dental restoration with osseointegrated implants is performed three to six months after fibula free flap reconstruction. In patients with significant subcutaneous adipose tissue in their fibula free flap skin paddle, thinning of the fat is usually performed simultaneous with placement of the implants. Partial or total hardware removal is sometimes necessary in order to place the osseointegrated dental implants.

### 2.3. Orbital Floor Defects

Our experience suggests that, when supported by a soft tissue free flap, the orbital floor can usually be successfully reconstructed with bone grafts or alloplasts, even when postoperative radiation therapy is administered. Many surgeons, however, feel that bone grafts are relatively more resistant to radiation-associated complications than alloplasts are [24]. In a recent review of orbital floor reconstruction for trauma, Kirby et al. [25] found that autologous bone reconstructions were more likely to be complicated by orbital dystopia and enophthalmos compared to titanium mesh and porous polyethylene reconstructions, possibly due to increased difficulty in shaping the reconstructed orbital floor, irregular thickness, and unpredictable resorption. Obviously, alloplastic materials have the advantage of being available in virtually unlimited quantities and carry with them no donor site morbidity.

When using the fibula osteocutaneous free flap for reconstructing hemipalatomaxillectomy and bilateral palatomaxillectomy defects that include resection of the orbital floor, some soleus or flexor hallucis longus muscle can be included with the fibula free flap to support a bone graft or alloplastic orbital floor reconstruction. A double-barreled design to reconstruct both the floor and the hard palate is possible but challenging because of the limited space in the mid-face [26]. Patients who have had a complication following nonvascularized reconstruction can usually be reconstructed secondarily with a bony free flap, such as the fibula, scapula, and serratus anterior with rib, or radial forearm osteocutaneous free flaps.

In patients who have had orbital wall reconstruction, periodic light perception and visual acuity checks are necessary to rule out optic nerve injury or globe compression. Any signs of decreased vision should prompt an immediate ophthalmology consult and potential return to the operating room. Extraocular movements should be assessed as well, and a forced duction test at the conclusion of the surgery should always be performed.

### 2.4. Orbital Exenteration Defects

The primary goal of reconstruction is to line the orbital cavity with durable tissue as well as to exclude the nasal or sinus cavities when the medial or inferior orbital wall has been removed and protect the brain when the orbital roof has been removed. Additionally, the patient's desire for prosthetic rehabilitation should be considered when planning the reconstruction. A deep orbital cavity facilitates prosthetic fit while a bulky flap that sits flush with the face or bulges outward may not securely hold a prosthesis without osseointegrated implants. A bulky flap may also cause the prosthesis to protrude unnaturally.

Healing by secondary intention and granulation may be the simplest treatment after tumor resection. The entire process can take months and requires daily wound care with wet to dry dressings. When completely healed by secondary intention, the orbital cavity is only slightly shallowed with granulation tissue but allows easy inspection for local tumor recurrence. Wound closure can be accelerated by using a meshed or unmeshed split thickness skin graft to line the orbital cavity. Similar to healing by secondary intention, the split thickness skin grafting of the orbital defect also results in excellent visualization of the orbital cavity. Due to their thinness, skin graft reconstructions usually result in a deep orbital cavity that provides an excellent fit for an orbital prosthesis if one is desired by the patient. When there is no history of prior radiation and no postoperative radiation is planned, secondary intention and skin grafting even on bare orbital bone are usually successful methods for addressing the standard orbital exenteration wound.

If radiation is planned after surgery or if radiation has been given previously, more durable, well-vascularized lining of the orbital cavity with soft tissue rather than just spontaneous epithelialization or skin graft coverage is necessary to avoid chronic bone exposure and osteoradionecrosis. Among local pedicled flaps used, the most common ones are the temporalis muscle flap and temporoparietal fascia flap. The temporalis muscle flap, based on the anterior and posterior deep temporal arteries arising from the internal maxillary artery is thin enough to permit a reasonably secure fit for orbital prostheses, even without osseointegrated implants but results in a depression at the donor site, which may be cosmetically unfavorable in many patients [27]. The temporoparietal fascia flap covered by a split-thickness skin graft may be preferable in that there is no donor site deformity. In both cases, reach of these two flaps may be limited and it may be necessary to remove the lateral orbital wall in order to cover the entire orbital cavity. Large scalp or forehead flaps should be avoided when other reconstructive options are available because of their donor site disfigurement.

In extended orbital exenteration, the size of the cavity usually necessitates soft tissue coverage larger than what local or regional flaps can provide [28]. An exception is the resection of the lateral orbital wall alone, which is usually inconsequential from a reconstructive standpoint. The orbital cavity may still be reconstructed with a skin graft or regional flap. In addition, limited defects of the medial orbital wall can often still be reconstructed with a temporalis muscle flap. For all other defects, reconstruction should be performed with a microvascular free flap. A multitude of soft tissue free flaps are satisfactory for reconstruction of the extended orbital exenteration cavity.

Our preference is to reconstruct the cavity with a radial forearm fasciocutaneous free flap in cases where the bony resection is limited [27]. This flap provides an adequate amount of tissue with relatively little bulk in nonobese patients to accommodate an orbital prosthetic without revision surgery. In cases where the bony resection is more extensive a larger, bulkier flap is preferred. The RAM or the ALT free flaps are good choices in this situation [27]. Both flaps may be designed such that muscle tissue obliterates the sinuses and creates a seal over any exposed dura, preventing infection with sinonasal bacterial flora. Bulkier flaps such as these are required to restore mid-facial volume and preserve cheek contour.

**Figure 3:**
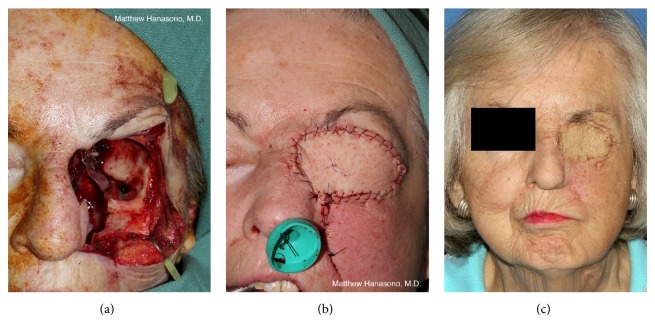
Orbitomaxillectomy defect following tumor removal (a). An anterolateral thigh free flap is used to close the defect and isolate the orbital cavity from the sinonasal cavities (b). A soft nasal trumpet is used to stent the nasal cavity open. Postoperative appearance (c).

Orbital exenteration performed in concert with a suprastructure maxillectomy (i.e., orbitomaxillectomy) adds another reconstructive priority: closure of the nasal cavity to prevent the escape of air and nasal drainage. Because the formation of a sinonasocutaneous fistula along the suture line between the medial flap (or native cheek skin) and the nose can be difficult to treat, every effort should be made to obtain a secure closure. While thin fasciocutaneous flaps that result in a concave orbital cavity are beneficial for patients with isolated orbital exenteration defects that wish to have an orbital prosthesis, for patients with orbitomaxillectomy defects, bulkier free flaps that obliterate the orbital cavity in order to minimize the chance of a fistula as well as maintain cheek contour are indicated. The rectus abdominis free flap, harvested as a myocutaneous flap or as a muscle-sparing variant (i.e., muscle-sparing rectus abdominis free flap or deep inferior epigastric perforator flap), is suitable and can be tailored to the specifics of the defect in terms of size, volume, and desired pedicle length. The ALT free flap also works well for orbitomaxillectomy reconstruction (Figure 3). A myocutaneous ALT free flap, incorporating some or all of the thickness of the vastus lateralis muscle, can be used when the thigh is thin and has insufficient adipose tissue to restore cheek contour.

Defects involving both an orbital exenteration defect and a palatomaxillectomy defect are best reconstructed with flaps that allow for multiple skin paddles to close the three defects (external orbital skin, nasal lining, and palatal coverage) separately allowing for airtight skin-to-skin and skin-to-mucosa closure. Both the RAM free flap and ALT free flap are usually good choices here as well, again tailoring the flap design to include more or less muscle based on the extent of the defect. In cases where the pedicle of the ALT free flap only gives rise to a single cutaneous perforator, a portion of the skin paddle can be deepithelialized to reconstruct two or more surfaces. It is the author's preference to usually leave a “raw” (noncutaneous) flap surface facing the nasal cavity and allow it to spontaneously remucosalize.

### 2.5. Complications

In our experience, the success rate of free flaps used in mid-facial reconstruction is as high as free flaps used to reconstruct other head and neck defects [17]. Because of the greater distance from the cervical recipient blood vessels, there is the potential for pedicle length to be inadequate. In such instances, interposition vein grafting is preferable to performing anastomoses under tension. Also, care must be taken to make an adequately large subcutaneous tunnel for the pedicle to reach the neck without compression.

Specific to maxillary and orbital reconstruction is the potential for oronasal (i.e., palatal) fistulae and sinonasocutaneous fistulae, usually near the medial canthus [17]. Closure along palatal and facial suture lines must be multilayered and meticulous. Nasal obstruction should be avoided as air under pressure can cause wound breakdown along lateral rhinotomy incisions and medial orbital incisions. In our experience, late occurring fistulae in irradiated patients rarely heal spontaneously and usually require another free flap for closure.

With regard to orbital reconstruction, there is a potential for graft or implant exposure along the orbital rim when tissues are thin and poorly vascularized, especially when incisions are placed over bony prominences or titanium hardware. In such cases, consideration should be given to placing flap tissue between bone grafts, implants, or hardware and the cheek or eyelid skin or to replacing the skin with a flap skin. Also, accurate positioning of the reconstructed orbital walls is critical to avoiding orbital content entrapment, enthophthalmos or exophthalmos, vertical dystopia (eyes at different levels), or even blindness due to elevated intraocular pressures or impingement of the optic nerve if grafts and implants are placed too far posteriorly in the region of the orbital apex.

## 3. Mandibular Reconstruction

Reconstruction of the mandible can be quite complex and time-consuming following resection of cancers. Mandibular defects frequently involve composite tissues including oral mucosa and soft tissue structures, mandibular bone, and, in some cases, external skin. Nevertheless, many advances have been made in mandibular reconstruction in the past two decades, including the development of vascularized bone flaps, low profile, high tensile strength reconstruction plates, the ability to restore dentition with osseointegrated implants, and the incorporation of computer-aided design (CAD) and computer-aided manufacturing (CAM) into the surgical planning.

Goals of mandibular reconstruction include reestablishing the shape of the lower third of the face, creating a surface for mastication or dental restoration, preventing deviation of the jaw leading to malocclusion, maintaining free movement of the temporomandibular joint, and providing a stable wound that does not result in an orocutaneous fistula. In addition, mandibular reconstruction should not result in tethering the tongue in a way that affects speech or deglutition or introduce redundant tissue that can obstruct the airway or compromise oral hygiene. The reconstruction must also be reliable and long lasting. Mandibular reconstruction that results in early fracture or extrusion of bone flaps or grafts, as well as reconstruction plates, results in a situation that is frequently even more difficult to treat than the initial defect [29].

### 3.1. Regional Anatomy

The mandible provides the bony support for the lower third of the face and bears the lower teeth. Each side of the mandible consists of a lateral horizontal portion, called the body, a perpendicular portion, called the ramus, an alveolar process along the upper border of the mandible, which bears the teeth, a condyle, which forms the temporomandibular joint with the temporal bone, and a coronoid process, which is a triangular bony projection, anterior to the condyle. The paired mandibular bones join anteriorly in the midline at the symphysis. The portion of the body just lateral to the symphysis is referred to as the parasymphyseal region. The body and the ramus meet in a region termed the angle of the mandible (also known as the gonial angle). There are two foramina on each side of the mandible: the mandibular foramen on the deep side, which is penetrated by the inferior alveolar nerve, a branch of the mandibular division of the trigeminal (V) nerve, and the mental foramen on the superficial side, from which the mental nerve, a continuation of the interior alveolar nerve, emerges inferior to the second premolar tooth and supplies sensation to the lower lip.

Many muscles attach to the bony mandible. Anteriorly, the paired genioglossus muscles attach to the inner surface of the mandible at the mentum, lateral to the lower part of the symphysis. Immediately inferior to the attachment of the genioglossus muscles is the attachment point of the paired geniohyoid muscles. The mylohyoid, digastric, and the superior pharyngeal constrictor muscles also attach to the inner surface of the mandible along its inferior, inner border. Loss of the anterior mandible, therefore, can have profound effects on swallowing and protection from aspiration due to posterior displacement of the tongue and limited laryngeal elevation, which can also contribute to airway obstruction. Because of this, close airway monitoring and strong consideration for prophylactic (usually temporary) tracheostomy are indicated. Patients need to be counseled regarding potential impairment of swallowing and chronic aspiration following anterior mandibular resection. Speech can also be affected and the tongue will have limited protrusion.

The muscles of mastication insert along the posterior mandible. The temporalis muscle inserts mostly onto the coronoid process, while the masseter muscle inserts broadly along the lateral surface of the mandibular ramus and posterior body. The lateral pterygoid muscles attach to the condylar neck on each side, while the medial pterygoid muscles, which serve to depress the mandible and open the mouth, insert on the medial surface of the angle. Loss of the posterior portion of the mandible, therefore, results in impaired mandibular movement on the affected side. Even with reconstruction, mouth opening and closing will be dependent upon the actions of the contralateral muscles of mastication. Tension from the medial and lateral pterygoid muscles will tend to rotate the mandible toward the side of the resection.

### 3.2. Reconstructive Approach

Options for treating mandibular defects include performing no reconstruction with primary closure of the oral soft tissues to themselves, reconstruction with metal plates, nonvascularized bone grafts, osteomyocutaneous pedicled flaps, soft tissue pedicled or free flaps, and osseous or osteocutaneous free flaps.

### 3.3. Mandibular Reconstruction with Reconstruction Plates

Mandibular reconstruction with a reconstruction plate that spans a segmental bony mandibular defect was a more popular technique prior to the development of microvascular free bone flaps. Some centers continue to utilize reconstructive plates when a patient is deemed unsuitable for a prolonged operative procedure involving free tissue transfer or when microvascular expertise is unavailable. However, experience has shown that such reconstructions are at high risk for complications, including plate fracture, and/or exposure, either intraorally or through the skin of the cheek or chin [30].

To help decrease the rate of exposure, many surgeons have combined reconstruction plates with a pectoralis major muscle or myocutaneous pedicled flap or a soft tissue free flap. However, Wei et al. [29] still reported a complication rate of 69% in patients reconstructed with soft tissue free flaps, primarily the anterolateral thigh fasciocutaneous free flap and a reconstruction plate, in a series of 80 patients. Plate exposure was the most common complication, followed by soft tissue deficiency, deformity of the lateral face, intraoral contracture, trismus, and osteoradionecrosis. Thirty-one percent of the patients with complications ultimately underwent a secondary salvage procedure with a fibula osteocutaneous flap.

Overall, complication rates associated with a reconstructive plate and soft tissue flap are reported to be between 21 and 87 percent [30, 31]. Anterior defects are associated with a higher rate of plate extrusion than lateral defects as are defects in patients undergoing radiation treatment or with a history of prior irradiation. In addition, larger defects result in significantly higher failure rates than smaller defects.

Even when patients are reasonable candidates for a free flap, some surgeons advocate plate or plate and soft tissue flap reconstruction in patients with advanced cancers and a limited life expectancy, since surgery is usually shorter and recovery is usually faster. However, this approach must be carefully considered on a case-by-case basis, because the incidence of plate-related complications is high. In addition, the results are rarely ideal due to persistent contour deformity and malocclusion. Secondary salvage of such complications with vascularized bone flaps can be performed but tends to be much more difficult than if it is performed at the time of the resection due to more challenging dissection of recipient vessels and greater difficulty restoring accurate occlusion secondary to postoperative and radiation therapy-associated scar contracture.

### 3.4. Nonvascularized Bone Grafts

Autogenous bone grafts can be used for mandibular reconstruction. The bone is revascularized by a process of creeping substitution. Sources of cortical bone graft include iliac crest, split calvarium, and rib. Nonvascularized bone grafts may be used in defects less than about 5 cm long. High failure rates are frequently seen in longer segments and in anterior defects. Preoperative or postoperative radiation therapy is a contraindication due to high rates of extrusion, resorption, and infection, and, therefore, use of nonvascularized bone grafts for mandible reconstruction is usually restricted to patients with benign disease or who require mandibular surgery for orthognathic rather than oncologic indications. Metallic mesh or Dacron trays filled with cancellous bone chips have also been used for limited defects. This technique is associated with a high rate of extrusion and bone graft dissolution, especially in cancer patients, and has generally fallen out of favor.

### 3.5. Vascularized Bone Flaps

Mandibular reconstruction with vascularized bone flaps transferred by microsurgical anastomosis should be considered the gold standard in oncologic reconstruction [32, 33]. Use of vascularized bone flaps is associated with early bony union, generally within six weeks. Vascularized bone flaps demonstrate very little bony resorption. Unlike nonvascularized bone grafts, bone flaps can be used to reconstruct large segmental bone losses and can tolerate radiation therapy without resorption, fracture, or extrusion. The commonly used bone flaps may be harvested with a cutaneous or muscular component that allows for simultaneous soft tissue reconstruction. Some flaps can be harvested simultaneously with oncologic resection or cervical vessel dissection by a second team to save time. Some vascularized bone flaps can reliably accept osseointegrated implants, which require a minimum of 6 to 7 mm of bone height for stable placement [34].

Pedicled bone flaps, such as the pectoralis major muscle with rib or sternal bone and the trapezius muscle with scapula, have also been described [35]. The pectoralis osteomyocutaneous flap can be used for anterior defects and the trapezius osteomyocutaneous flap for lateral defects. The lack of reliability, particularly of the distal flap that supplies the bone, limited ability to shape and configure both the soft tissue, and the bony flap components to fit the defect, restricted reach, and limited availability of bone make these two flaps secondary options after free bone flaps. Their use is primarily of historic significance.

#### 3.5.1. Fibula Free Flap

**Figure 4:**
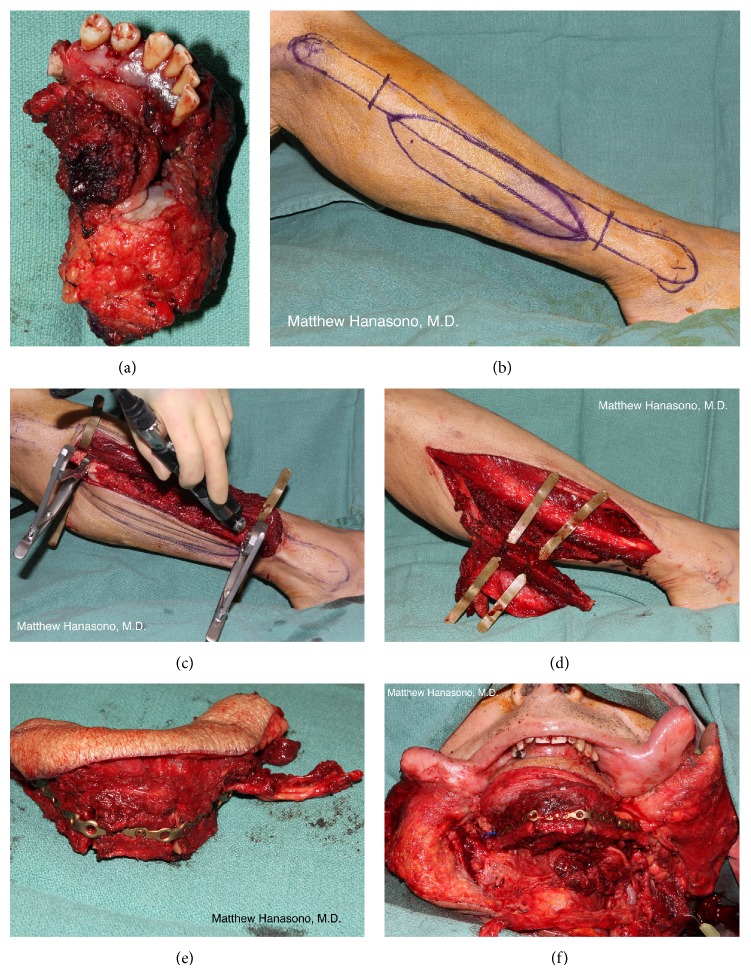
Composite anterior mandibular resection for a large invasive floor of mouth cancer (a). Skin markings for a fibula osteocutaneous free flap (b). Approximately 5 to 7 cm of proximal and distal bone are left* in situ* (c). Osteotomies for shaping the fibula can be made prior to or after pedicle division (d). Fibula osteocutaneous free flap after rigid fixation with a titanium reconstruction plate (e). Flap inset (f).

The fibula osteocutaneous flap is probably the most frequently used choice for mandibular reconstruction (Figure 4) [32, 33]. The fibula bone is primarily an ankle stabilizer and provides the origin for several muscles of the lower leg but is expendable provided that the distal several centimeters of the bone, including the lateral malleolus, are spared. A 22 to 25 centimeters segment of fibula bone may be harvested in the adult patient, permitting reconstruction of near-total mandibular defects with a single flap.

The vascular supply of the fibula free flap is the peroneal artery and vein. It is important to examine both lower extremities and palpate for dorsalis pedis and posterior tibial pulses preoperatively [36]. In addition to pathologic conditions, it is important to rule out the possibility of peroneal arteria magna, an anatomic variant where the peroneal artery is the dominant arterial inflow to the distal lower extremity and a fibula free flap is contraindicated [37]. A patient with findings consistent with arterial insufficiency or venous stasis may not be a candidate for a fibula free flap. When the pedal arteries are not palpable or the circulation to the lower extremity is questionable, additional studies may be required, such as conventional, magnetic resonance, or computed tomographic angiography of the lower extremities, prior to performing fibula free flap harvest.

The choice of leg is based on the anticipated side of the recipient blood vessels and expected need for extra- or intraoral lining. The author usually prefers to use the leg that is contralateral to the side of the recipient blood vessels when intraoral lining is needed based on the location of the septocutaneous perforators that travel along the posterior-lateral border of the fibula in its native position on the leg. The flap is oriented so that the pedicle is on the lingual side, to minimize external compression and allow plate placement on the lateral aspect of the fibula and, for posterior defects, is usually placed posteriorly close to the branches of the external carotid artery and the internal jugular vein.

Osteotomies may be completed while the flap is left* in situ* or during the inset of the flap into the mandibular defect. Performing the osteotomies while the pedicle is still attached to the leg has the advantage of minimizing ischemia time. Also, any injuries to the pedicle can be identified well in advance of revascularization, an advantage that is perhaps most important when rigid fixation and skin paddle inset are performed prior to the microvascular anastomosis. Other surgeons prefer to perform osteotomies after the pedicle is divided due to increased freedom of movement, potentially avoiding traction injury to the pedicle blood vessels.

The author prefers to use a locking titanium reconstruction plate to secure the osteotomized fibula to the remaining native mandibular segments. Some surgeons have had success with using smaller, very low profile miniplates. Such plates have the advantage of permitting fine adjustments to the final shape of the reconstructed mandible while locking reconstruction plates are considered to possess superior stability and are able to tolerate higher loads.

In certain cases, a double-barrel approach to mandibular reconstruction is used to increase bony height [38]. In this technique, reconstruction proceeds in the usual manner but the distal portion of the fibula is turned back 180 degrees onto the proximal fibula for additional height of the reconstructed mandible to more closely approximate the height of the normal dentulous mandible. The double barrel technique is best suited for reconstruction anterior mandibular defects, as the normal height of the mandible is greater in this region. Laterally, the width of a single fibular segment closely approximates the height of the native mandible [22]. When a single width of fibula is used, the fibula is aligned with the lower border of the mandible, rather than the alveolus, in order to achieve the best possible external contour.

Malocclusion following mandibular reconstruction may still not occur infrequently. Whenever possible, the mandible is preplated prior to mandibular resection so that the reconstruction can be designed to maintain the spatial orientation of the native mandible. When preplating is not feasible due to an exophytic tumor, pathologic fracture, or prior resection, use of an external fixator can be considered. More recently, use of computer planning and rapid prototype modeling has helped improve outcomes, particularly when preplating is not feasible [39–41].

#### 3.5.2. Iliac Crest Free Flap

The iliac crest free flap provides a generous amount of cortical and cancellous bone for mandibular reconstruction. The deep circumflex iliac vessels comprise the vascular pedicle of the iliac crest free flap and demonstrate consistent anatomy, reasonable length (average of eight to ten centimeters), and appropriate vessel diameter (average of two to three millimeters) for microsurgical application. The blood supply of the iliac crest bone flap is robust, incorporating both nutrient perforators and periosteal vessels allowing the flap to tolerate multiple osteotomies.

The iliac crest bone may be harvested as a full-thickness bicortical or as a partial-thickness unicortical (inner cortex) bone flap. Unicortical bone flaps are associated with a superior donor site appearance and theoretically less donor site morbidity but less bone stock [42]. The natural curved contour of the bone is often considered ideal for lateral mandibular reconstruction. Reconstruction of anterior defects usually requires an osteotomy. The bone stock, particularly when harvested full-thickness, reliably accommodates osseointegrated dental implants.

The flap may be harvested as a bone-only flap or with an associated skin and/or muscle paddle for reconstruction of composite defects. The skin paddle, which is nourished by several perforators arising from the deep circumflex iliac vessels, may be as wide as 9 to 12 cm and still be closed primarily in most patients. Previously, osteocutaneous iliac crest free flaps included harvesting a cuff of external oblique muscle, internal oblique muscle, and transversalis fascia with the skin paddle, but in recent years perforator dissection has been more commonly performed, resulting in a less bulky soft tissue component [43]. Even when the skin island is dissected as a perforator flap without a muscular component, iliac crest osteocutaneous free flaps may be excessively bulky in some patients and can require substantial primary or later revisionary thinning or use of a second, thinner flap for soft tissue reconstruction. Alternately, a separate internal oblique muscle paddle, based on the ascending branch of the deep circumflex iliac artery, which arises within 1 cm of the anterior superior iliac spine, can also be harvested for reconstruction of composite defects and provide a thin layer of soft tissue coverage.

The donor site, while hidden in clothing, may result in a contour deformity and/or hernia. Gait abnormalities also occur not infrequently. Harvesting the bone flap as a split-cortical flap decreases the morbidity of the flap by preserving hip contour, minimizing gait disturbances, and providing better support for the abdominal viscera resulting in a decreased risk for hernia. Meticulous hemostasis and closed suction drainage of the donor site are recommended as there is significant potential for donor site hematomas and seromas. Obesity is a relative contraindication to performing reconstruction with the iliac crest free flap due to difficulty in flap dissection and increased risk for postoperative donor site hernias.

#### 3.5.3. Scapular Free Flap

Another alternative for mandibular reconstruction is the scapular free flap. The scapula flap has traditionally been based on the circumflex scapular artery. The length of the pedicle can be increased several centimeters by including the more proximal subscapular vessels. The subscapular vessels are also of larger caliber than the circumflex scapular vessels, which may be an advantage in performing the microvascular anastomosis.

The bone may be harvested from either the lateral or the medial edge of the scapula. The lateral scapular bone flap, based on the vertically oriented parascapular branch of the circumflex scapular artery, has a shorter vascular pedicle but is thicker. The medial scapular bone flap, based on the horizontally oriented cutaneous scapular branch of the circumflex scapular artery, is thinner but associated with a longer pedicle and minimal disturbance of the teres major and minor muscles and the glenohumeral joint, resulting in less postoperative shoulder stiffness. Approximately 10 to 14 centimeters of linear bone may be harvested from either the lateral or medial aspect of the scapula.

A skin paddle, based on a cutaneous branch of the circumflex scapular artery, can be harvested with the osseous portion of the flap if needed. For larger defects, a chimeric flap utilizing the subscapular regional blood supply can be harvested to include a scapular or parascapular skin paddle and the latissimus dorsi (with or without an overlying skin paddle) and serratus anterior muscles [44–46]. The serratus anterior can be harvested with a rib to allow for a second bony reconstruction. Furthermore, a thoracodorsal artery perforator skin flap can also be harvested rather than a latissimus dorsi muscle or myocutaneous flap along with other flaps arising from the subscapular axis. The potential configurations are numerous.

Another variation of the scapular osseous flap involves utilizing the angular branch of the thoracodorsal artery [46]. This branch usually arises from the latissimus dorsi branch of the thoracodorsal artery and lies within the submuscular fat pat beneath the superior edge of the latissimus dorsi and teres major muscles. It enters the scapular bone near the inferior angle or tip of the scapula. Basing the scapular flap on this branch allows for a longer pedicle, up to 17 cm if dissected to the axillary artery, and reliably supplies the medial, lateral, and angular portions of the scapular bone. The author has used it in anterior mandibular reconstructions in patients who are not candidates for fibula free flap reconstruction [45]. In this case, the curved shape of the scapular tip nicely restores the shape of the anterior mandible without the need for osteotomies for shaping. However, a major disadvantage of the scapular bone, regardless of its pedicle blood supply, is that it is often quite thin and does not consistently provide enough bone stock for osseointegrated implant placement.

Although a potentially reliable donor site, the scapula flap requires careful planning for positioning the patient during flap harvest, preparation of recipient vessels, microvascular anastomosis, and flap inset. The location of the scapula makes it very difficult to perform a two-teamed approach for harvesting the flap and preparation of the recipient site. Patients may note a degree of shoulder stiffness and limited abduction following the harvest of the scapula flap. Because of this, physiotherapy should routinely be part of the postoperative course.

#### 3.5.4. Radial Forearm Free Flap

For soft tissue reconstruction of the head and neck, the radial forearm fasciocutaneous free flap has a reliable, long (up to 20 centimeters) vascular pedicle. The flap is thin and pliable and can be made sensate by neurorrhaphy of the lateral antebrachial cutaneous nerve to the inferior alveolar nerve. This flap can also be designed as an osteocutaneous flap by inclusion of the anterior (volar) cortex of the radial bone. Up to 14 centimeters of unicortical radius nourished by periosteal branches from the radial artery may be harvested for selected osseous defects. However, the use of the osteocutaneous radial forearm free flap is typically not a first line option due to the limited thickness of the bone that may be harvested without disturbing the structural mechanics of the hand and the risk for radial bone fracture in the forearm after harvest [47].

From a third to a half of the radius may be harvested for limited bony defects. Care must be taken not to detach the flexor pollicis longus muscle attachment to the radius, although a portion of the muscle is included in the flap as a muscle cuff that contains the radial artery perforators nourishing the bone. The distal limit of the bony flap is the insertion of the brachioradialis tendon and the proximal limit is the pronator teres muscle insertion. Osseointegrated implant placement in the radial bone flap has been accomplished but it is generally accepted that these are less reliable than those performed in the other bone flaps.

The donor site morbidity following the harvest of an osteocutaneous radial forearm free flap can be significant. Tendon rupture, carpal tunnel syndrome, and a significant motor weakness have been reported [47]. Radial bone fracture is estimated to occur about 15 percent of the time. Some surgeons suggest prophylactically plating the radius in the same setting as harvesting the flap. Nonetheless, the potential for chronic hand weakness or pain coupled with the thinner donor bone stock of the radial forearm osteocutaneous flap decreases enthusiasm in using this flap as a primary choice in reconstruction of load-bearing segmental mandibular defects, particularly in the anterior mandible.

### 3.6. Reconstructive Algorithm

Concerning the various types of flap choices for osseous mandibular reconstruction, each flap varies in terms of pedicle length, skin paddle, flap length, ability to accept osseointegrated implants, and patient morbidity. The iliac crest and fibula free flaps most reliably accept osseointegrated implants consistently due to their thickness [48]. The scapular free flap offers numerous soft tissue reconstruction options when harvested as part of a chimeric free flap with other skin and muscle components supplied by the subscapular arterial axis and may provide adequate bone stock for osseointegrated implants in select patients. The skin paddle of the radial forearm free flap is highly reliable and thin, making inset easier. In terms of vascular pedicle length, the radial osteocutaneous free flap has the longest available potential vascular pedicle. This option may be appealing in the patient with an absence of satisfactory local recipient vessels. However, this flap is limited by the very thin bone stock it provides and the risk for radial bone fracture at the donor site, as mentioned. The fibula free flap provides the longest amount of bone, has a good pedicle length and caliber, and, when dissected appropriately, has a reliable skin paddle. Based on its versatility, as well as acceptable donor site morbidity, the fibula free flap is the preferred method of mandibular reconstruction for those defects in which a vascularized bone flap is indicated at most centers, with other free flap choices being secondary and based on soft tissue needs, donor site availability, risk for complications, and patient positioning.

The specific reconstructive technique chosen should also depend on the location of the mandibular defect. Although several defect classification systems have previously been described, the decision-making process can be simplified by considering whether the mandibular defect is anterior, lateral, or posterior. Anterior defects involve the region of the parasymphysis and symphysis, anterior to the first bicuspid. The lateral defects occur posterior to the canine, encompassing some or all of the mandibular body, and extend to the angle of the mandible or the mid-ramus but spare enough of the ramus and condyle to preserve joint movement and allow for titanium plate fixation. Posterior defects are those that involve the condyle and can be limited to the ramus or extend anteriorly to encompass the body up to the parasymphysis. Therefore, the presence of the condyle and upper ramus of the mandible differentiates lateral defects from posterior defects.

#### 3.6.1. Anterior Mandibular Defects

The anterior mandible may be involved with floor of mouth and anterior tongue cancers, as well as occasionally with lower lip cancers by direct extension. As with all cancers invading the mandible, a preoperative computed tomography (CT) scan is requisite, and evidence of cortical invasion will be an indication that a segmental mandibulectomy will be required. Note that tumors that abut the mandible but do not invade the cortex are often treated with a marginal mandibulectomy (removal of the upper or lingual bony cortex of the mandible) and can usually be covered with a soft tissue pedicled or free flap alone.

Any defect that includes the anterior mandible should be reconstructed with vascularized bone whenever possible (Figure 4). Failure to reconstruct the anterior mandible results in the so-called “Andy Gump” deformity, a condition that is disfiguring and may be functionally problematic in terms of mastication, pooling of saliva, loss of oral competence, and even airway support. It is in this region that the mandible forms a curve and provides projection to the lower face, and, therefore, bony reconstruction is required to maintain facial symmetry.

**Figure 5:**
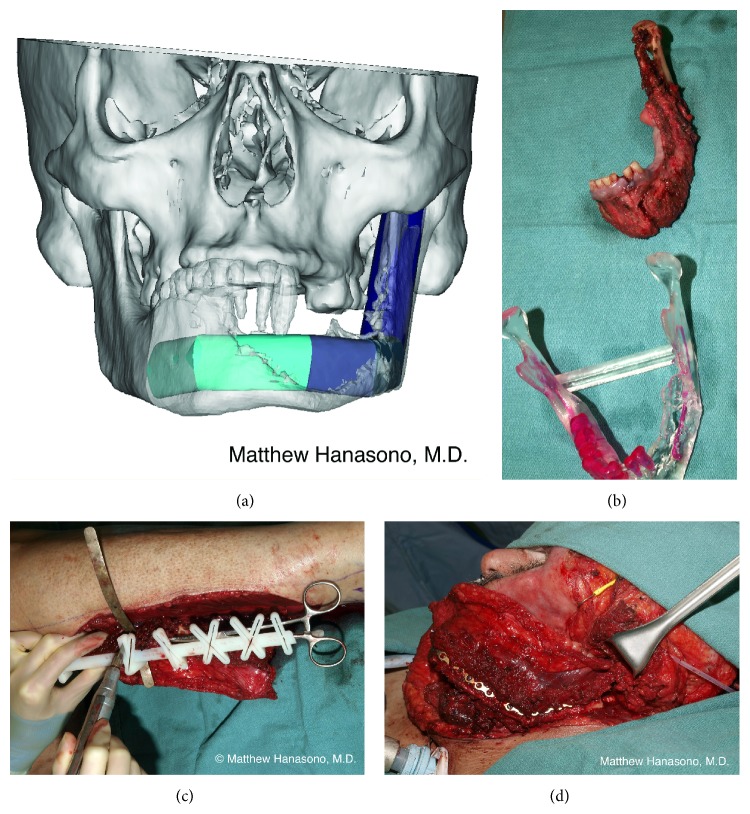
Virtual plan for mandibular reconstruction using computer-assisted design software (figure courtesy of Medical Modeling, Incorporated, Golden, CO) (a). Comparison of mandibular specimen to rapid prototype model (b). Computer-generated slotted cutting guide used to help make osteotomies in a fibula osteocutaneous free flap (c). Flap inset (d).

The linear bone of the fibula will require one or more wedge-shaped osteotomies to restore the curvature of the mandible. Whenever possible, it helps to prebend the titanium reconstruction plate to the native mandible prior to the resection. The reconstruction plate is used as a guide for shaping the fibula free flap (or other bone flap). The simplest technique for planning the osteotomies is by cutting a paper or plastic template, such as a sterile paper ruler, to fit the shape of the titanium plate. More recently, computer-generated cutting guides have become available that facilitate making precise osteotomies at the angles dictated by the preoperative plan created using computer-aided design software (Figure 5).

The mandibular height in the dentulous patient is greater in this region than the lateral mandible, averaging 33.5 mm in males and 31.1 mm in females [22]. By comparison, the width of the lateral surface of the fibula averages 17.9 mm in men and 13.1 mm in women [22]. Hence, a double-barrel configuration may be desirable in the anterior mandible. In such cases, the lower “barrel” of the fibula should ideally project anteriorly several millimeters further than the upper “barrel” to maintain both chin projection and occlusion. If a single-barrel configuration is used, the fibula is inset so that it matches the occlusion of the maxilla. In edentulous patients, the mandible will tend to overrotate and anterior projection can be substantially increased. In such cases, the fibula will need to be set back considerably (1 to 2 cm or more) if there are no plans for dental restoration to limit mandibular excursion and avoid the appearance of a severe prognathia.

#### 3.6.2. Posterior Mandibular Defects

Posterior mandibular resections may result following the surgical treatment of many different types of cancers, including retromolar trigone cancers, tonsillar and lateral pharyngeal wall cancers, and base of tongue cancers. A number of primary bone tumors that develop from impacted third molars can require resection of this part of the mandible as well. In some cases, large buccal, maxillary, and soft palate cancers can extend inferiorly and involve the posterior mandible. Finally, deeply invasive skin cancers, parotid cancers, and temporal bone cancers can require removal of the posterior portion of the mandible, although, in such cases, there will not necessarily be a mucosal defect.

**Figure 6:**
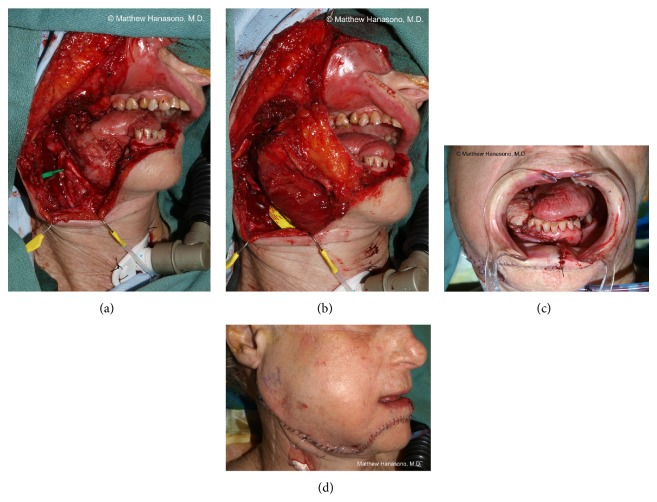
Posterior mandibular defect following tumor resection (a). Anterolateral thigh myocutaneous free flap inset (b). Skin paddle to close oral defect (c). External appearance (d).

It is established that reconstruction with vascularized bone flaps is preferred for defects that include the anterior mandible to minimize complications and to maximize oral function. Reconstruction of the posterior mandible, specifically those in which the condyle and sufficient subcondylar ramus to accommodate secure fixation with titanium hardware is lacking, is more controversial. Use of soft tissue alone with reasonable results has been reported [49, 50]. In these reports, soft tissue free flaps such as the anterolateral thigh (ALT) free flap and the rectus abdominis myocutaneous (RAM) free flap were found to allow single flap closure with adequate tissue bulk to replace the missing mandible and associated soft tissues as well as providing a skin paddle to resurface the oral mucosal defect (Figure 6).

Soft tissue reconstruction can result in acceptable cosmetic appearance, speech, and swallowing function [49, 50]. Other advantages include potentially reduced operative time compared to bony flap harvest and shaping, faster recovery, and a low complication rate. Furthermore, in cases where the posterior mandible has been resected, bony reconstruction may not have significant advantages as the ipsilateral masticatory musculature is generally not reconstructable, nor is there perfect replacement for the condylar joint (see below).

When soft tissue is used, the flap must be adequately bulky to prevent substantial deviation of the mandible toward the resected side. Anticipating soft tissue atrophy, especially when postoperative radiation is administered, some volume overcorrection is desirable. A pectoralis major myocutaneous pedicled flap may also give satisfactory results in posterior mandibular reconstruction. However, the pectoralis major myocutaneous pedicled flap can be limited in reach and arc of rotation for very high or posterior defects. Because late contraction of the pedicle may also cause a descent of the bulk of the flap toward the neck or result in limited neck mobility, the author considers pectoralis major myocutaneous flaps a second choice to a bulky soft tissue free flap. Whether bony or soft tissue reconstruction is utilized, there will be risk for malocclusion due to loss of the condyle and temporomandibular joint disruption. Nonedentulous patients tend to experience fewer problems with malocclusion since they can use their remaining teeth to guide their jaws into the proper occlusal relationship.

Although satisfactory functional and cosmetic results for posterior mandible reconstruction with soft tissue free flap reconstruction can be achieved, bony reconstruction is probably still best for highly functional patients who are able to tolerate more extensive operations and bony free flap harvest. In our experience, occlusion is usually, although not always, superior with bony reconstruction [50]. As the defect extends more anteriorly, both the improved occlusion and cosmetic appearance owing to maintaining a better-defined jawline favor bony reconstruction.

#### 3.6.3. Condylar Defects

The temporomandibular joint is a diarthrodial joint in which the condyle of the mandible is separated from the glenoid fossa of the temporal bone by a cartilaginous disc. Derangement of the joint or removal of the disc or condyle can lead to pain, instability, or trismus. Movement of the temporomandibular joint is complex and is comprised of both a rotational and a gliding process. Numerous methods of condylar and temporomandibular joint reconstruction have been described. Because of its complexity, no uniformly satisfactory technique has been found and reconstruction of this region remains a subject of controversy.

Reconstruction of the rest of the mandible is addressed whenever appropriate with osseous or osteocutaneous free flap reconstruction as described above. Tumors of the superior ramus rarely invade the temporomandibular joint, and if enough of the upper ramus can be spared, the optimal reconstruction would be to fixate the bony free flap to the mandibular remnant. However, the superior cut in posterior segmental mandibulectomies often results in minimal subcondylar bone being left behind, making stable fixation with rigid titanium plates difficult or impossible. In many cases, the condyle is, therefore, completely removed. In addition, all masticatory muscles are detached from the mandible and the ligamentous support of the joint is disrupted.

Reconstruction of the condyle with titanium prostheses has been described. While some success has been noted, complications including infection, plate fracture, extrusion, and erosion into the middle cranial fossa are not uncommon. For this reason, such prostheses, even those with a silicone cap, have largely been abandoned in most centers. This technique should generally be avoided in cancer patients, who are at elevated risk for complications, particularly because of the frequent need for radiation therapy.

Reconstruction with costal cartilage grafts has also been described. The costochondral graft appears to be most useful in isolated condylar defects, providing a soft articulating surface with the glenoid fossa. In larger defects, such as is the case in most oncologic resections, a nonvascularized cartilaginous graft lacks adequate length and stability and is prone to resorption, particularly in cases where postoperative radiation therapy is given.

Temporomandibular joint reconstruction with plating of the native condyle onto a vascularized bone flap as a graft has been described by Hidalgo [51]. In a series of 14 patients who underwent hemimandibular resection, the resected condyle was mounted on a free vascular bone flap as a nonvascularized graft. Some condylar resorption was noted in a few patients but this did not correlate with a decrease in function. It was felt that use of the native condyle aided in accurate free bone flap placement. No recurrences stemming from the transplanted condyle were observed. Wax et al. [52] attempted this technique in 2 patients, one of whom had displacement of the condyle out of the glenoid fossa and the second had poor cosmetic and functional outcomes. Experience with this procedure has not otherwise been described in the literature.

Replacement of the condyle with the fibular head or a contoured, rounded fibular end has also been used by several centers, including our own. A pseudoarthrosis is thought to form. The end of the bony flap can be anchored by a suture that extends from the fibular periosteum or a hole drilled in the bone to the cut end of the pterygoid tendon or a hole drilled into the lip of the glenoid fossa to minimize drift of the bony flap end out of the temporomandibular joint. The end of the fibular bone flap is rounded with a cutting burr and fits into the glenoid fossa.

#### 3.6.4. Lateral Mandibular Defects

Lateral mandibular defects usually arise from extension of lateral tongue and floor of mouth cancers, buccal cancers, and submandibular gland cancers. Osteoradionecrosis may also be an indication for lateral mandibular resection, since this region of the mandible frequently receives substantial radiation during the treatment of oropharyngeal cancers, while the condyle, ramus, and parasymphyseal regions are relative spared.

Missing lateral segments are usually bridged with bone. Bony reconstruction allows for very accurate results, including restoration of preoperative occlusion. Here too, vascularized bone flaps are usually preferred, but nonvascularized bone grafts may be considered for very small defects in healthy wound beds that have neither been radiated nor are expected to be radiated postoperatively. Some surgeons feel that reconstruction plates can be used to bridge lateral defects, however, because there is a significant risk of plate fracture over time, as mentioned above, this type method should be avoided if at all possible. In general, reconstruction of such defects with bony free flaps is usually straightforward as few, if any, osteotomies are required. Also, the height of the midpoint of the mandibular body averages only 25.7 mm in males and 24.5 mm in females, so double-barreled fibular reconstruction, which is more time-consuming and complicated, is usually unnecessary [22].

In combined lateral and posterior defects (i.e., defects in which the condyle and subcondylar region cannot be spared that also extend anteriorly to involve the mandibular body), as well as in patients who are not good candidates for bony free flap reconstruction, soft tissue reconstruction can be performed. If lateral segments are not reconstructed with bone, the posterior mandibular segment bearing the condyle should be removed to prevent eventual erosion of the bone medially into the oral cavity caused by the continued pull of the pterygoid muscles. In many cases, satisfactory functional and aesthetic results can be achieved with a pedicled or free soft tissue flap for lateral and posterior-lateral defects, just as in purely posterior defects as described above. However, the more anterior the defect extends, the more noticeable a contour deformity will be due to the absence of the lower mandibular bony margin and a visible “step-off” where the native mandible ends. Additionally, the more anterior the defect extends, the greater the potential for malocclusion is as the mandible rotates toward the side of the defect due to the pull of the contralateral pterygoid muscles.

### 3.7. Massive Defects

In selected cases, head and neck reconstruction may require a second microvascular free flap or regional pedicled flap for closure of extensive defects to achieve the best functional and aesthetic results [53, 54]. Such reconstructions may be limited by the availability of recipient blood vessels. Strategies such as the use of chimeric flaps (e.g., the scapular bone/latissimus dorsi muscle/serratus anterior muscle flap), vein grafting to more distant recipient vessels, such as the transverse cervical or contralateral neck vessels, and the “piggy backing” technique, where one flap is anastomosed to the pedicle of another flap, may be required [55].

One commonly used combination is the use of a soft tissue skin or myocutaneous flap for intraoral reconstruction, such as when there is a substantial glossectomy defect, and a bony flap such as the fibula to restore the mandible. A thin-pliable flap, such as the radial forearm flap, is recommended for reconstruction of a hemiglossectomy defect, while a bulkier ALT or RAM free flap is recommended for near-total or total glossectomy defects. While the use of a large skin paddle from a single fibula osteocutaneous free flap may close the oral wound, a two-flap procedure is advocated to maintain tongue mobility in patients who can tolerate an extended surgery and have a reasonable oncologic prognosis in order to optimize the functional outcome.

**Figure 7:**
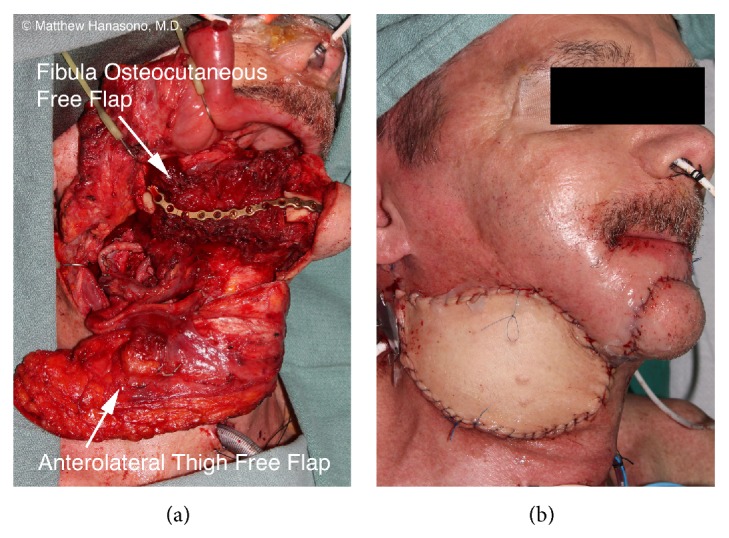
A fibula osteocutaneous free flap for mandibular and intraoral reconstruction and an anterolateral thigh free flap for external neck skin reconstruction (a). Completed reconstruction (b).

When there is a through and through defect, an osteocutaneous bone flap is usually used with its skin paddle providing the intraoral lining and a soft tissue free flap providing the external coverage (Figure 7). Another option is the use of a chimeric osteocutaneous scapular-latissimus dorsi (or thoracodorsal artery perforator) free flap or 2-skin paddle fibula osteocutaneous free flap (when more than one set of cutaneous perforating blood vessels are present). The pectoralis major myocutaneous pedicled flap may also be used to reconstruct the external cheek defect, although this flap is usually reserved as a “lifeboat” in case of complications such as a flap loss or fistula.

Occasionally, a maxillary and mandibular defect is encountered and may be another indication for a two-free flap reconstruction. In such cases, a myocutaneous ALT or rectus abdominis flap is used for unilateral maxillary and buccal reconstruction and a fibula osteocutaneous free flap is used for mandibular reconstruction. However, limited mandibular defects combined with palatomaxillary defects can often be addressed with a single large ALT or RAM free flap when the mandibular defect does not involve the anterior mandible, such as might arise from a retromolar trigone tumor that extends superiorly or a posterior palatal tumor that extends inferiorly.

### 3.8. Osseointegrated Implants

Dental rehabilitation may be achieved by the use of fixed or removal prostheses that are retained by osseointegrated dental implants. The efficacy of this technique has been demonstrated in the noncancer edentulous population. Use of osseointegrated dental implants requires adequate bone stock and a well-vascularized recipient tissue bed with stable soft tissue coverage. Placement of osseointegrated implants into the remaining edentulous native mandible or free vascularized bone flaps has been very successful in carefully selected patients [56]. Implants must be surrounded by a minimum of 1 mm of healthy bone. The fibula and iliac crest free flaps offer the best bone stock for osseointegration, while the scapula and radial forearm do so less reliably. Implant failure is increased in patients who smoke or have had radiation therapy or poor oral hygiene with dental infections.

The choice of timing for placement of osseointegrated implants, either primarily at the time of microvascular free bone flap reconstruction or secondarily after initial healing, has been completed depending on a number of factors including whether the resected lesion was benign or malignant, status of adjacent skin and soft tissues, plans for postoperative radiation, and patient motivation. Some authors regard radiotherapy to host bone as a contraindication to implant placement. Urken et al. [57] reported a 92-percent success rate of endosteal dental implants in vascularized mandibular reconstructions. The rate of implant success in which implants placed were irradiated postoperatively was 86 percent; implants placed into previously irradiated bone had a 64 percent success rate. Other issues that are debated include whether implants should be placed prior to or after radiation therapy, the maximum radiation dose associated with acceptable risk for implant placement, and the length of time that should elapse after radiation treatment prior to implant placement in delayed cases.

### 3.9. Complications

Specific to bony free flap mandibular reconstruction, malunions and nonunions are rare. If they occur, they can many times be successfully treated with debridement of the bone edges and rigid fixation, provided the free flap remains viable. Fistulae can occur and should be treated promptly with irrigation and debridement if there is purulent fluid in the vicinity of the pedicle, anastomosis, or cervical recipient vessels to prevent thrombosis or vascular rupture.

We allow patients who undergo fibula free flap reconstruction to ambulate as early as postoperative day 2, even in a splint, with weight bearing as tolerated on the affected limb. While not ambulating, we require the patient to keep the donor limb elevated at all other times, whether in bed or in a chair, to facilitate skin graft healing. Donor site complications occur in about 30 percent of patients, the vast majority of which are managed conservatively [58]. It may take up to 3 months for patients to return to their baseline of ambulatory status following fibula free flap harvest. Long-term complications are relatively uncommon but may include persistent weakness, ankle instability, great toe contracture, and decreased ankle mobility.

## 4. Oral Cavity and Pharyngoesophageal Reconstruction

The oral cavity is the most common site for squamous cell carcinoma of the head and neck. The tongue and floor of the mouth are the most common sites for primary cancers in the United States whereas buccal cancer is most common in some regions in Asia. The organs in the oral cavity, particularly the tongue, play critical roles in speech and swallowing. The base of the tongue is more important for swallowing function, whereas the oral tongue is more important for speech and food manipulation. Proper reconstruction of these vital organs in the oral cavity is necessary to maintain the airway, avoid fistulae, restore speech and swallowing function, and improve quality of life and self-image.

Pharyngoesophageal defects are most commonly the result of a total laryngopharyngectomy for squamous cell carcinoma in the laryngeal region or hypopharynx. Other etiologies include benign strictures, pharyngocutaneous fistulas, and thyroid cancer by direct extension. Since radiotherapy has become the primary treatment for early stages of squamous cell carcinoma in these regions, many pharyngoesophageal defects are the results of salvage laryngopharyngectomy following chemoradiation failure, making reconstruction more challenging. The goals of reconstruction are to provide alimentary continuity, protection of important structures such as the carotid artery, and restoration of speech and swallowing.

### 4.1. Regional Anatomy

The oral cavity is bounded by the lips anteriorly and the base of tongue and soft palate posteriorly. Subsites of the oral cavity include the floor of mouth, oral tongue (anterior two-thirds of the tongue, up to the circumvallate papillae), buccal mucosa, hard palate, mandibular and maxillary alveolar ridges, and retromolar trigones. The oral tongue is a critical structure for speech articulation and manipulating food. The hypoglossal (XII) nerve innervates all the muscles of the tongue except for the palatoglossus, which is innervated by the vagus (X) nerve. The facial (VII) nerve, via the chorda tympani, and the lingual (V3) nerve are responsible for taste and sensation of the oral tongue, respectively. Squamous cell carcinomas arising from the mucosa are the most common type of cancer affecting the oral cavity. Salivary gland cancers, arising from the submandibular, sublingual, and minor salivary glands, are the next most common.

The pharynx is divided into the nasopharynx, oropharynx, and hypopharynx. The nasopharynx extends from the skull base to the level of the soft palate. Most cancers of the nasopharynx are treated with combined radiation and chemotherapy and surgical defects in this region are rare. The oropharynx extends from the soft palate to the hyoid bone. The soft palate, tonsils, tonsillar pillars, base of tongue, and the pharyngeal walls at this level are all considered parts of the oropharynx. The soft palate prevents nasal regurgitation while the base of tongue and pharyngeal walls, which contain constrictor muscles, play a critical role in deglutition. The hypopharynx extends from the hyoid bone to the cricopharyngeus muscle, which is the most important component of the upper esophageal sphincter. The piriform sinuses, postcricoid area, and posterior pharyngeal wall comprise the hypopharynx. The hypopharynx may be the site of primary cancers, again most commonly squamous cell carcinomas, or may be involved in laryngeal cancers by direct extension.

### 4.2. Oral Cavity Defects

#### 4.2.1. Floor of Mouth Defects

If no bone is exposed and there is no communication with the neck, floor of mouth defects can be allowed to mucosalize spontaneously or skin grafted. Partial skin graft loss is common for oral cavity reconstruction; however, areas of loss usually remucosalize spontaneously.

**Figure 8:**
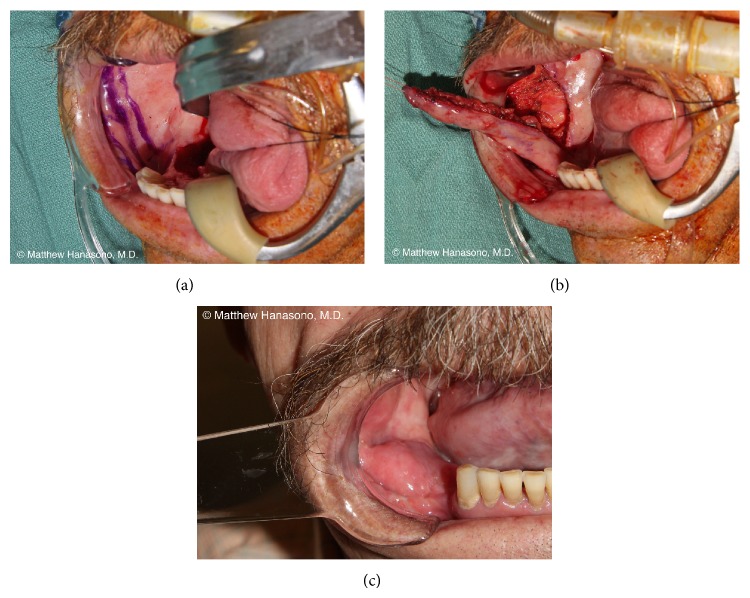
Design of a facial artery musculomucosal pedicled flap for a lateral floor of mouth and mandibular gingival defect (a). The flap is elevated and includes a portion of the buccinator muscle and the facial artery, which is deep to the muscle (b). Postoperative appearance (c).

Small defects of the floor of mouth with bone exposure can be repaired with a facial artery musculomucosal (FAMM) flap (Figure 8). The FAMM flap is based on the facial artery and includes a portion of the buccinator muscle in addition to the buccal mucosa and is usually useful for small defects up to about 2 cm in width that enable primary closure of the donor site [59–61]. The blood supply to the FAMM flap is the facial artery. When elevating a FAMM flap, a small amount of buccinator muscle is included in the flap, along with the buccal mucosa and the facial artery. Venous drainage depends mainly on the buccal venous plexus. The FAMM flap can be superiorly based on the angular artery to repair palatal defects but needs to be inferiorly based on the main facial artery in order to be rotated to the floor of the mouth. Prior to elevation, a handheld Doppler ultrasound is used to trace the course of the facial artery. The width of the flap is limited by the amount of laxity in the buccal mucosa that allows primary closure of the donor site, usually around 2 cm.

The submental flap is another local option that can be harvested with a width of 4 to 6 cm, depending on the redundancy of the submental skin, while allowing primary closure of the donor site. This flap is supplied by the submental branches of the facial artery and vein [62–64]. The anterior belly of the digastric muscle is usually included to ensure adequate perfusion since the small arterial and venous blood supply, which is often not visualized within the submental fat, is deep to the muscle about 70 percent of the time and superficial 30 percent of the time. The pivot point is roughly at the angle of the mandible. Both the submental and FAMM flap may be unavailable as options following neck dissection in which the facial artery is ligated.

The pedicled pectoralis major myocutaneous (PMMC) flap or pectoralis major muscle flap covered by a skin graft can be also used for extensive floor of mouth as well as many other oral cavity reconstructions. These flaps are based on the thoracoacromial artery and can reliably cover most oral cavity defects. The skin paddle of the PMMC flap is reliable when designed to include adequate cutaneous perforators [65]. As mentioned above, disadvantages of the pedicled pectoralis major flap include limited reach, neck contracture due to fibrosis of the proximal muscle, and, if a lot of proximal muscle is included in the flap, an unsightly bulge in the neck. Despite these drawbacks, pectoralis major flaps are still frequently used in patients who are poor free flap candidates, as an additional flap in conjunction with a free flap to reconstruct massive defects or as a secondary option in the event of a free flap failure. In contrast, for patients who are in satisfactory medical condition with a reasonable functional and oncologic prognosis, free flaps are the gold standard.

The radial forearm fasciocutaneous (RFF) free flap is useful for moderate to large floor of mouth defects since it is thin and pliable thus preventing compromised speech or swallowing due to excess bulk or tethering of the tongue. The RFF is based on the radial artery and is rapidly harvested with a long pedicle thereby facilitating head and neck reconstruction. Drawbacks of the RFF flap are decreased circulation to the hand, risk for tendon exposure due to incomplete skin graft take and a relatively unfavorable donor site appearance.

Several steps are taken to minimize donor site morbidity following RFF flap harvest. The author prefers to use the venae comitantes as vein outflow rather than the cephalic vein. In many patients, the cephalic vein is far away from the radial artery. Including the cephalic vein requires a flap design that is more dorsal, resulting in a more noticeable donor site scar. Designing the skin paddle more proximally to avoid the wrist crease when the dimensions of the defect permit also decreases morbidity by not having watches or bracelets rub against the skin graft postoperatively. A suprafascial harvest, in which the fascia investing the forearm muscles and tendons is spared, may decrease donor site morbidity without compromising flap viability [66, 67]. The superficial radial sensory nerve more easily avoided by staying in the suprafascial plane, but, nevertheless needs to be identified and consciously preserved. The venae comitantes are usually no larger than 1.5 mm in diameter before they converge; therefore, the vein is usually taken above the convergence of the venae comitantes, where the diameter is greater than 2.5 mm in most cases.

An alternative to the RFF free flap is the ulnar artery perforator (UAP) flap [68]. The UAP flap relies on discrete perforators that usually arise 8 cm or more proximal to the hamate bone. Tendon exposure following UAP flap is more limited than in the RFF. The donor site is more hidden and small defects can be closed primarily when the arm skin is lax, as in the elderly. Ulnar forearm skin is usually less hairy than radial forearm skin and just as thin. There are usually one to three true perforators arising from the ulnar artery and its accompanying venae comitantes.

The disadvantage of the UAP flap is that the pedicle is shorter, usually 4 to 5 cm long. However, in most head and neck reconstructions, the recipient vessels are within short reach. Therefore, a long vascular pedicle is not needed. Also, dissection along the ulnar nerve can be tedious and transient paresthesias can be experienced by the patient postoperatively. Because of the potentially lower donor site morbidity, the UAP flap is often selected over the radial forearm flap when a long pedicle is not needed.

The distal border of the flap is usually proximal the wrist crease to avoid tendon exposure. Dissection is usually performed under tourniquet control. Suprafascial dissection is performed until the perforators are seen medially and laterally where the fascia is incised and subfascial dissection is carried out. The perforators are small, and, therefore, meticulous dissection is required. The ulnar nerve is carefully separated from the ulnar artery and vein and retraction of the nerve should be avoided. The medial antebrachial cutaneous nerve is included in the flap for sensory reinnervation.

For floor of mouth resections that result in substantial submandibular dead space, slightly bulkier flaps, such as the ALT free flap, are useful. The ALT free flap is particularly useful in head and neck reconstruction because it can be transferred either as a fasciocutaneous flap or as a myocutaneous flap depending on the reconstructive needs. When harvested as a fasciocutaneous free flap, it is usually intermediate in thickness between the RFF flap and the RAM flap. The RAM flap is based on the deep inferior epigastric vessels and is too bulky in most patients with isolated floor of mouth defects. Although the bulk of the flap can be decreased by harvesting it as a fasciocutaneous flap based on the deep inferior epigastric perforator (DIEP) vessels, even without the rectus abdominis muscle the DIEP flap is usually thicker than the ALT free flap.

#### 4.2.2. Buccal Mucosa Defects

The goal of reconstruction for defects involving the buccal mucosa is to prevent cicatricial trismus. Primary closure can be used for small defects, and split- or full-thickness grafts can be used for moderate ones. For defects involving the majority of the buccal mucosa, a thin, pliable fasciocutaneous free flap such as the RFF or UAP flaps are needed to prevent scar contracture from limiting mouth opening. The ALT flap may also be used in thin patients and may have the advantage of decreased donor site morbidity as compared to forearm flaps. The ALT free flap can also be thinned considerably at the time of surgery, taking care not to injure the perforator blood supply and the subdermal vascular plexus of the flap, as well as reduced secondarily with suction-assisted lipectomy. Buccal mucosa resections that result in through and through cheek defects often require reconstruction with flaps that can either be folded on themselves, deepithelializing a portion of the flap to allow wound closure at the flap margin, or harvested with dual skin paddles. ALT and RAM free flaps can be designed with more than one skin paddle, allowing separate reconstruction of the buccal mucosa and external cheek skin with a single flap. The RFF and UAP flaps can often be safely split when there are multiple branches of the pedicle vessel supplying the proximal and distal skin paddle, but their size usually required them to be used for smaller cheek defects.

#### 4.2.3. Tongue Defects

Partial tongue defects can be closed primarily or with full thickness skin grafts to prevent graft contracture. If primary closure or a graft is likely to result in significant tongue tethering, a flap is usually indicated for closure. In practical terms, flaps are commonly required for defects approaching half the tongue and larger. Additionally, a through and through defect communicating with the dissected neck is usually best addressed with a flap to decrease the risk of fistula.

**Figure 9:**
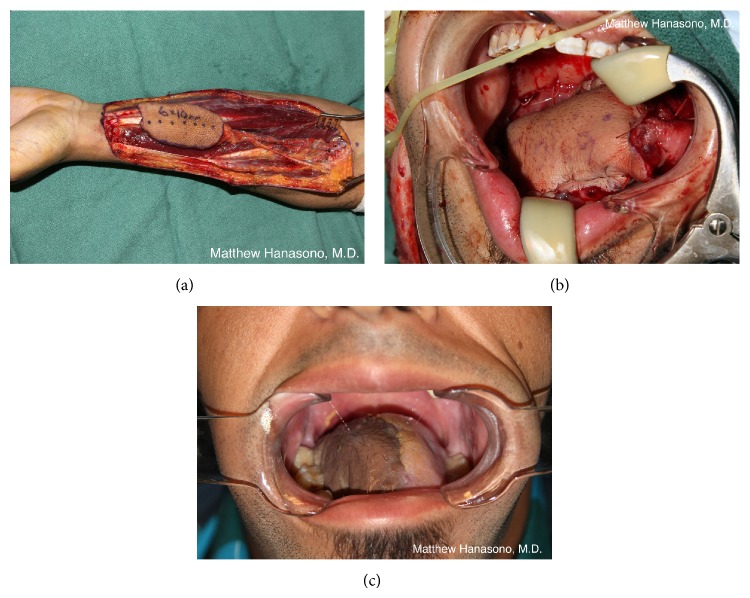
Radial forearm free flap harvest for a right hemiglossectomy reconstruction (a). Flap inset (b). Postoperative appearance (c).

For hemiglossectomy defects, a thin, pliable flap is needed to preserve tongue mobility, although a small amount of bulk is also needed to obliterate the oral cavity dead space with the mouth closed and not create a funnel for secretions to drain directly into the larynx. The goal is to allow the residual tongue to contact the premaxilla and palate for speech articulation, as well as to be able to sweep and clear the oral cavity, and move food and secretions from anterior to posterior [69–71]. Here again, most surgeons prefer the RFF free flap oriented such that the distal end of the flap is used to reconstruct the anterior portion of the tongue (Figure 9). Adequate flap width is needed to prevent tethering the tip of the tongue to the floor of mouth and to recreate a sulcus. Bulkier free flaps or the PMMC flap can also be used in more extensive resections; however these options typically have inferior results in terms of speech and swallowing.

In addition to the RFF or UAP free flaps, a regional option for partial and hemiglossectomy reconstruction is the supraclavicular artery island flap (SCAIF), provided the flap is long enough, based on the patient's anatomy, not to result in tethering of the reconstructed tongue [72–77]. The SCAIF is an axial pattern flap based on the supraclavicular artery, which is usually a branch of the transverse cervical artery that originates from the thyrocervical trunk, although on rare occasions it can arise from the suprascapular artery. The supraclavicular artery is a thin diameter vessel that can be reliably found in the supraclavicular triangle, between the clavicle, sternocleidomastoid and trapezius muscles, from which it travels toward the acromion.

On the day of surgery, the pedicle location is confirmed using Doppler ultrasound. A suture can be fixated at the pivot point and the radius of rotation and skin paddle length can be estimated and marked out with a surgical marker. An elliptical skin paddle is then designed according to the defect and also includes an additional 2 to 3 cm of skin medial to the pedicle origin. The skin paddle may be up to 6 to 8 cm wide and still be closed primarily in some patients, depending on the skin laxity of the shoulder area. Lengthwise, the flap can extend up to 3 cm distal to the acromion. The length of the SCAIF skin island can be increased by delaying the flap, elevating it but returning it to its original position without rotation for a period of about 7 days.

The skin incision is carried down through the skin and subcutaneous tissue through the fascia of the anterior deltoid muscle. The SCAIF is then dissected from distal to proximal in a subfascial plane. As the flap is harvested more proximally, the underside of flap is checked with a handheld Doppler to confirm inclusion of the supraclavicular artery. The pedicle can also be visualized in the medial third of the flap by transillumination of the skin. Proximally, a 1 to 2 cm cuff of subcutaneous fat surrounding the pedicle is preserved to avoid injury to the source vessel. Several sensory nerves are encountered during the dissection and can be divided to increase the arc of flap rotation. Once the flap dissection is completed lateral to the pedicle, the remaining medial skin incisions are performed to complete the skin island paddle.

The strategy for reconstruction following near total and total glossectomy is different than for hemiglossectomy [78–81]. A bulkier flap is required to reconstruct the greater volume of resection and flaps such as the RAM or ALT are commonly used. Swallowing and speech outcomes are better when the flap can be made convex into the oral cavity. To do so, it is helpful to design the flap to be somewhat wider than the oral defect, at least 8 to 9 cm in most cases, anticipating some atrophy of the flap with time, particularly if postoperative radiation will be administered. Additionally, many surgeons believe that laryngeal suspension using permanent sutures between the hyoid bone and mandible helps to prevent prolapse of the flap and also improves functional results by elevating the larynx. Laryngeal suspension from the mandible is performed with permanent circumhyoid sutures placed through drill holes on both sides of the mentum.

If at all possible, concave reconstructions creating a trough-like area should be avoided since pooling of secretions in the oral cavity can result in aspiration. Regardless, the patient should be counseled preoperatively about the possibility of unintelligible speech, inability to swallow, and chronic aspiration. The possibility of long-term tube-feeding and tracheostomy dependency following a total or subtotal glossectomy should always be discussed.

Although the complex motor function of the tongue cannot be restored with current reconstructive techniques, sensory reinnervation of free flaps is well documented [82, 83]. The RFF free flap can be made potentially sensate by coapting the lateral antebrachial cutaneous nerve to the stump of the lingual nerve using standard techniques. Similarly, the ALT and RAM free flaps can be made sensate by anastomosis of the lateral circumflex femoral and intercostal nerves, respectively, to the lingual nerve. Sensory recovery is variable. Thin free flaps, such as the RFF, have been shown to recover some sensation spontaneously even if nerve repair is not performed [84]. It remains unclear, however, whether the amount of sensibility typically recovered secondary to nerve repair actually translates into improved speech or swallowing [85].

Following hemiglossectomy reconstruction with the techniques described above, more than 90% of patients are able to resume an oral diet without the need for tube feeding, and most patients can tolerate a regular or soft diet, depending on their dental status. Tumor recurrence, a bulky flap, and aspiration can result in an inability to resume an oral diet. Aspiration occurs frequently in patients when the surgical resection extends to the epiglottis. With proper training by speech pathologists, most motivated patients can relearn how to swallow. Nearly all patients who have undergone a hemiglossectomy and reconstruction should be able to have their feeding tubes removed and speak intelligibly. Functional outcomes after total or subtotal glossectomy reconstruction remain disappointing. Overall, approximately half of the patients require partial or complete tube feeding.

### 4.3. Pharyngeal Defects

Many oropharyngeal cancers are more radiosensitive than oral cancers and radiotherapy is increasingly used as primary treatment in an effort to decrease morbidity secondary to surgical resection. Nevertheless, surgical resection is still indicated for extensive tumors, such as those that involve both the oral cavity and the oropharynx, and for recurrent cancers. The goals of reconstruction for the oropharynx include restoring continuity to the aerodigestive tract and replacing the volume of the tongue base in order to maintain swallowing function without aspiration.

**Figure 10:**
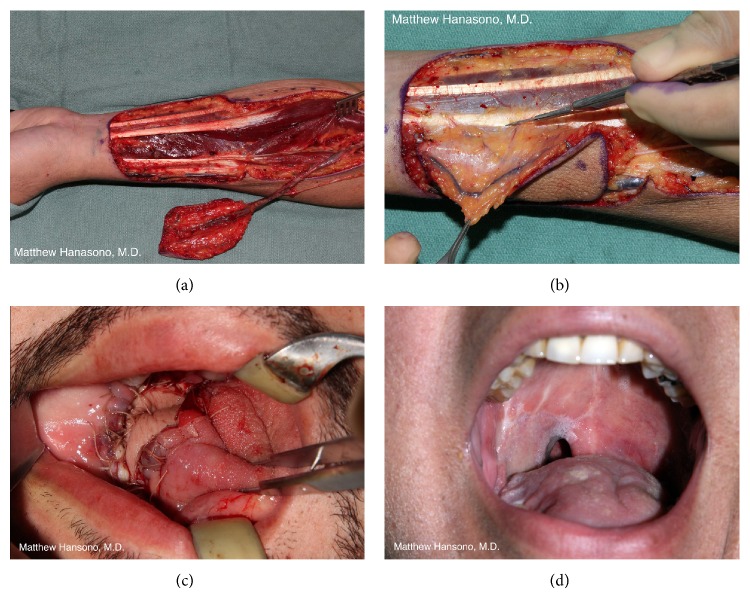
Radial forearm free flap harvest for a right oropharyngeal defect (a). To minimize donor site morbidity, a suprafascial harvest technique is used (b). Flap inset (c). Postoperative appearance demonstrating excellent mouth opening (d).

Defects of the tonsillar fossa and pharyngeal walls can be reconstructed with a skin graft or allowed to heal by secondary intention when they are small and superficial. Deep wounds, such as those that result in communication with the neck contents, require a flap for closure. These defects are typically closed with thin flaps such as the RFF or ALT (in nonobese patients) to avoid obstructing the airway or interfere with swallowing (Figure 10) [86]. Isolated base of tongue defects can sometimes be closed primarily. Partial defects, including those occurring in continuity with a tonsillar or retromolar trigone resection, are best reconstructed with a thin to moderate thickness fasciocutaneous free flap. Reconstruction of tongue base defects occurring as part of a near-total or total glossectomy requires bulkier flaps as discussed above.

Most tumors involving the hypopharynx, including both primary hypopharyngeal tumors and extensive laryngeal tumors, are malignant and are treated by laryngopharyngectomy. In such cases, reconstruction involves restoring a part or the entire circumference of the hypopharynx, sometimes extending to the cervical esophagus, thus restoring the continuity between the oral cavity and the distal esophagus for swallowing. Flaps are indicated for circumferential defects or for partial defects when primary closure results in a narrowed pharynx that will cause dysphagia or obstruction. Microvascular free flaps have replaced regional pedicled flaps, such as the PMMC flap, due to their lower fistula rates [87]. Free flap options include the jejunal free flap and fasciocutaneous free flaps, such as the ALT and the RFF free flaps.

The jejunal free flap is supplied by vascular arcades arising from the superior mesenteric artery and vein. A suitable segment located 20 to 30 cm from the ligament of Treitz is selected and the flap is isolated on a single arcade. The length of the jejunal segment required for the reconstruction is based on the pharyngeal defect, usually around 10 to 15 cm [88–91]. The flap can be split along the antimesenteric border to increase the diameter so that it is of suitable diameter to match that of the oropharynx and is inset into the defect in an isoperistaltic manner. Care must be taken to avoid redundancy as this may result in regurgitation and dysphagia. Warm ischemia time should be limited to less than 2 hours to avoid ischemia reperfusion injury. Intestinal continuity is restored in the abdomen and the wound is closed in a standard fashion after a feeding jejunostomy tube and a gastrostomy tube are inserted.

**Figure 11:**
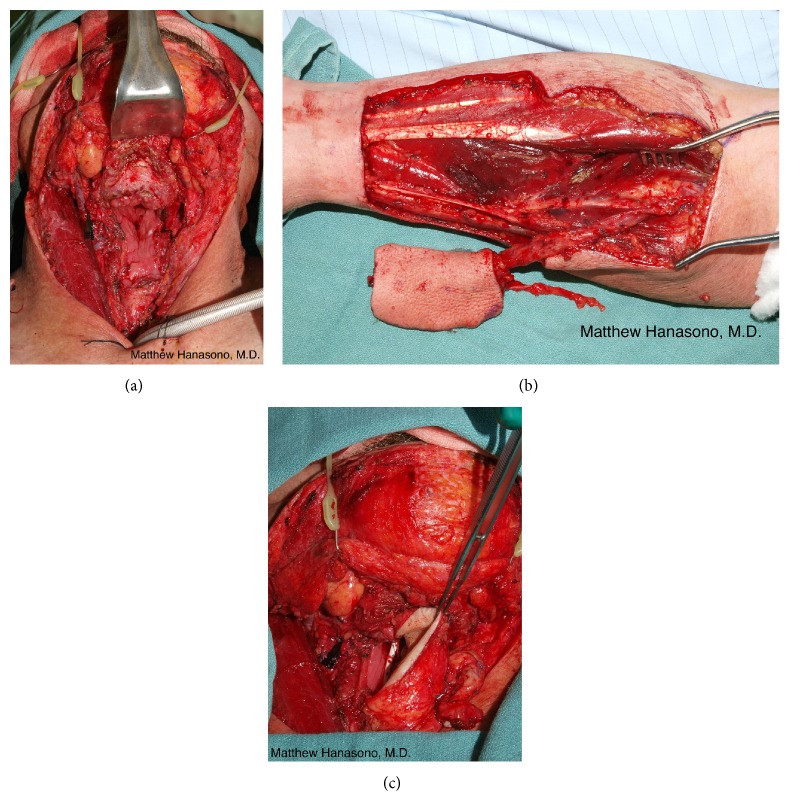
A partial circumference pharyngeal defect following a laryngopharyngectomy for recurrent laryngeal cancer (a). A radial forearm free flap was harvested for reconstruction (b). Flap inset over a nasogastric feeding tube (c).

The ALT free flap is another option for hypopharyngeal reconstruction [92–97]. To create a 3 cm diameter lumen, a 9.4 cm wide flap is required, based on the formula, circumference = *π* × diameter. Compared to the RAM free flap, the ALT free flap is usually thinner in most patients and thus more suitable for creation of a neopharyngeal conduit. The RFF free flap is also useful for hypopharyngeal reconstruction, particularly in partial circumference defects or in obese patients with excessive thigh thickness (Figure 11) [98, 99]. Some hypopharyngeal resections may spare a significant amount of the pharynx, and, occasionally, small or benign tumors can be resected with preservation of the larynx. In such cases, small fasciocutaneous flaps, such as the RFF free flap, are best suited to restoring pharyngeal continuity as a patch.

An advantage of the jejunal free flap is the avoidance of an additional suture line when reconstructing circumferential defects. The primary disadvantage of the jejunal free flap is the need for a laparotomy, which may result in postoperative ileus as well as the risks of anastomotic leakage of the repaired small intestine and potential late bowel obstruction due to adhesion formation. The ALT free flap is associated with minimal donor site morbidity but may be excessively thick in obese patients, although it usually tolerates aggressive thinning down to a thickness of around 0.5 cm, as long as the perforator and a cuff of subcutaneous tissue around it are carefully preserved. Both flaps are reliable with low rates of postoperative pharyngocutaneous fistula formation.

Radiation and chemotherapy are now used as primary therapy for laryngeal cancers in most centers. Thus surgical resections tend to most commonly be performed for cancer salvage, increasing the difficulty of reconstruction and the risk of wound healing complications, such as fistula [100]. In addition, previously irradiated neck skin tends to contract after skin flap elevation and may be at high-risk necrosis and wound dehiscence if closed under tension. In addition to potential exposure of the carotid artery and jugular vein, a wound dehiscence in the region of the tracheal stoma or pharyngeal closure could result in infection or fistula.

**Figure 12:**
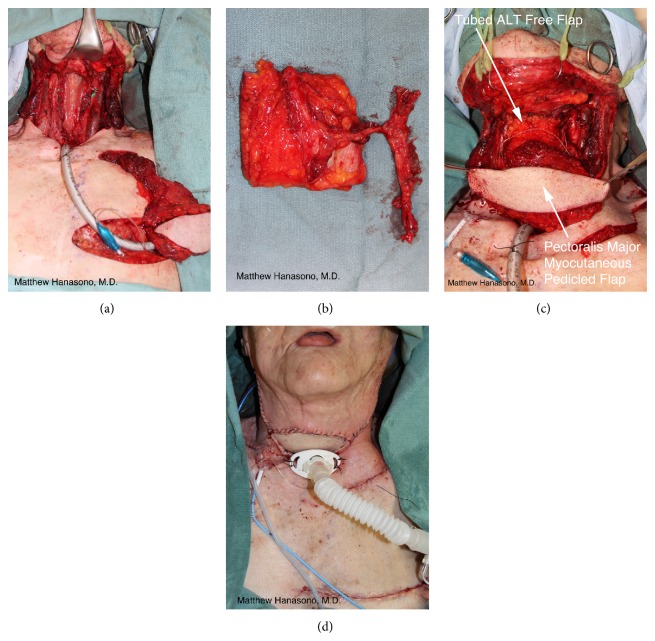
A total circumference pharyngeal defect following a laryngopharyngectomy for recurrent laryngeal cancer (a). A pectoralis major myocutaneous pedicled flap is dissected for neck skin coverage. The anterolateral thigh flap was dissected as a perforator flap and subsequently tubed (b). Inset of flaps (c). Postoperative appearance (d).

Reconstruction of the anterior neck skin often requires a second flap, either another free flap or a pedicled flap. The PMMC flap or pectoralis major muscle flap covered by a skin graft is frequently used to reconstruct the anterior neck skin (Figure 12). An elegant solution is to use a single flap to reconstruct both the pharynx and the anterior neck skin. The ALT free flap can often be designed with 2 skin paddles based on independent cutaneous perforating blood vessels that join together proximally within the main vascular pedicle, thus requiring only a single set of arterial and venous anastomoses to complete the reconstruction [101]. When more than one perforator is not available, the vastus lateralis muscle can be included with the ALT free flap and skin grafted to reconstruct the neck skin defect. Alternatively, the ALT free flap or other fasciocutaneous free flaps can be partially deepithelialized and a portion of the skin paddle can be used to reconstruct the neck skin defect.

An additional advantage of utilizing a second skin paddle from an ALT free flap for neck reconstruction is that it allows easy monitoring for microvascular anastomotic patency. When there is no external skin paddle, many surgeons utilize an internal Doppler (Cook-Schwartz Doppler) to monitor the buried free flap used for pharyngoesophageal reconstruction [102]. A third alternative is to design the ALT flap with two skin paddles, when there is more than one perforator, and temporarily leave the second skin paddle exteriorized through the neck incision, attached by its perforating blood vessel and a small amount of septal fascia [103]. This “monitoring” skin paddle is excised at the bedside prior to hospital discharge by ligating the perforator and amputating the skin paddle at the level of the neck skin. The small cutaneous defect can be left to heal secondarily or closed with one or two sutures under local anesthesia. The jejunal free flap is also often harvested longer than needed and a portion of the distal jejunum is exteriorized through the neck incision as a monitoring segment.

Vocal rehabilitation following laryngopharyngectomy can be accomplished by a number of methods, including use of an electrolarynx or a tracheoesophageal puncture (TEP) prosthesis. The TEP prosthesis is inserted into a surgically created hole between the posterior trachea and the cervical esophagus. If necessary, the hole can be placed through the reconstructive flap. The creation of the TEP can be performed at the time of reconstruction or in delayed manner, following flap healing. A one-way valve is part of the TEP prosthesis and allows shunting of air from the trachea to the pharynx and mouth for phonation when the tracheal stoma is occluded.

#### 4.3.1. Outcomes

In our group's early experience with 114 consecutive anterolateral thigh flaps used for pharyngoesophageal reconstruction, mean intensive care unit stay was 1.9 ± 2.2 days, and mean hospital stay was 9.0 ± 4.7 days [95]. Pharyngocutaneous fistulae and strictures occurred in 9% and 6% of patients, respectively. Ninety-one percent of patients tolerated an oral diet without the need for tube feeding. By comparison, in the jejunal flap reconstruction, the average hospital stay was 13 days and average intensive care unit stay was 3 days. The incidence of ileus and bowel obstruction was 9%, abdominal hernia, 6%, and anastomotic stricture, 19%. Overall, 65% of patients tolerated an oral diet without supplemental tube feeding, and 23% were partially tube-feed dependent, and 12% were totally tube-feed dependent. Fluent tracheoesophageal speech was achieved in 22% of patients who received a TEP. The quality of tracheoesophageal speech following jejunal flap reconstruction is usually characterized as “wet” or “cavernous” compared with that following a fasciocutaneous flap reconstruction or a total laryngectomy with primary pharyngeal repair.

### 4.4. Complications

Neck wound infection is the result of prolonged wound exposure and oral contamination during surgery. Many patients with oral cancer have poor oral hygiene because of pain. Prior chemoradiation and radiation therapy can also increase the risk of infection. Copious irrigation and obliteration of dead spaces are important for preventing postoperative wound infection. Once infection occurs, early drainage and thorough debridement and irrigation may enable primary wound closure over a drain; otherwise, the wound should be left open to allow further drainage. Serial operative irrigation and drainage procedures may be helpful in the worst cases. If there is dead space or vascular exposure occurs following debridement, pectoralis major muscle flap reconstruction of the neck may be indicated to protect the carotid artery, especially in patients who have had prior radiation therapy.

Wound dehiscences and fistulae can result from technical suturing errors, compromised tissue quality, most commonly due to prior irradiation and/or surgery, or wound infection. In our experience, pharyngocutaneous fistulas occur in about 9% of patients with ALT flap pharyngoesophageal reconstructions [97]. Fistula rates are similar in partial and circumferential reconstructions. There is the theoretical concern that the longitudinal seam of a tubed fasciocutaneous flap or two longitudinal suture lines in a partial defect might result in a higher incidence of fistula formation than the jejunal flap, which is a natural tube and only requires proximal and distal suture lines. However, in our group's experience, the fistula rate with the ALT flap is not significantly higher than with the jejunal flap. Fistulae usually develop between one and four weeks after surgery and manifest as leakage of saliva or liquids or, in some patients, as a purulent neck infection.

Once a fistula is identified, oral intake is withheld and local wound care is initiated. Small fistulas, in the absence of tumor recurrence or distal obstruction, usually heal spontaneously with conservative management. Therefore, a modified barium swallow (MBS) study is repeated about two weeks later to determine if the leakage has stopped. Larger fistulas or those with infection should be evaluated with CT to rule out abscess. The location of the fistula relative to the carotid artery and internal jugular vein is also evaluated. Any abscess around the carotid artery, especially in patients who have undergone previous radiation therapy should be drained and irrigated and consideration should be given to using a pectoralis major muscle flap to protect the carotid artery. Early intervention may achieve rapid healing and prevent life-threatening complications. With aggressive intervention, persistent fistulas are rare, therefore, if a fistula does persist, the patient should be evaluated for possible tumor recurrence and distal obstruction/stricture.

Anastomotic strictures usually occur at the distal anastomosis several months or years following reconstruction. Spatulation of the esophagus at the time of flap inset may reduce the risk of stricture formation. If a patient develops dysphagia after reconstruction, anastomotic strictures should be suspected and an MBS study was performed to confirm the diagnosis. Endoscopic balloon dilatation is our preferred initial treatment. Repeated dilatations may be required in some patients. In refractory cases, surgical enlargement of the strictured area may require an additional flap, such as the RFF free flap.

## 5. Conclusions

The 1990s saw a proliferation of free flap reconstruction for head and neck defects as they were demonstrated to be more reliable and result in superior functional and aesthetic outcomes compared to most prior techniques. In the past decade, algorithms were developed for flap selection based on evidence and experience, further improving outcomes and decreasing complications. Flaps are selected to minimize donor site morbidity, including perforator-based flaps, such as the anterolateral thigh free flap. Currently, advances in head and neck reconstruction are focused in further refinement, such as use of computer-assisted design and rapid prototype modeling to plan surgery. The future will undoubtedly bring further breakthroughs in reconstructive surgery in an effort to restore normalcy and allow for more complete oncologic resection with the goal of improving cancer cure rates and quality of life.
